# Replica Exchange Improves Sampling in Low-Resolution Docking Stage of RosettaDock

**DOI:** 10.1371/journal.pone.0072096

**Published:** 2013-08-29

**Authors:** Zhe Zhang, Oliver F. Lange

**Affiliations:** 1 Biomolecular NMR and Munich Center for Integrated Protein Science, Department Chemie, Technische Universität München, Garching, Germany; 2 Institute of Structural Biology, Helmholtz Zentrum München, Neuherberg, Germany; University of Michigan, United States of America

## Abstract

Many protein-protein docking protocols are based on a *shotgun* approach, in which thousands of independent random-start trajectories minimize the rigid-body degrees of freedom. Another strategy is enumerative sampling as used in ZDOCK. Here, we introduce an alternative strategy, ReplicaDock, using a small number of long trajectories of temperature replica exchange. We compare replica exchange sampling as low-resolution stage of RosettaDock with RosettaDock's original shotgun sampling as well as with ZDOCK. A benchmark of 30 complexes starting from structures of the unbound binding partners shows improved performance for ReplicaDock and ZDOCK when compared to shotgun sampling at equal or less computational expense. ReplicaDock and ZDOCK consistently reach lower energies and generate significantly more near-native conformations than shotgun sampling. Accordingly, they both improve typical metrics of prediction quality of complex structures after refinement. Additionally, the refined ReplicaDock ensembles reach significantly lower interface energies and many previously hidden features of the docking energy landscape become visible when ReplicaDock is applied.

## Introduction

Protein-protein interactions are one of the fundamental molecular mechanisms of life, and to investigate them it is important to know the atomic structures of the formed complexes. Since many proteins have multiple interaction partners, the number of protein complexes is far larger than the number of individually folded proteins. At the same time, the number of known complex structures is far lower than that of monomeric proteins, illustrating the experimental challenges involved in solving the structure of protein complexes [1]–[3].

Computational protein docking describes any *in silico* methodology for combining structural knowledge of individual protein components with general knowledge about protein complexes (often in form of a potential energy function) and, if available, sparse data of the complex [4]–[7]. Popular sources of sparse data include, cryo-EM, SAXS, NMR chemical shift perturbations or chemical crosslinking [1]–[3], [8], [9].

Currently, many popular docking programs employ a two-stage approach: First, conformational space is sampled broadly, keeping partner structures rigid. Second, structures are refined in one or multiple steps [10]–[14]. To account for possible side-chain or loop motion, many docking methods employ a low-resolution model during their initial rigid-body search to create the required level of softness without adding extra degrees of freedom [13], [15], [16]. Whereas some methods use solely a low-resolution representation, others refine structures in an all-atom representation, often allowing also side-chain and loop motion [17]–[20]. While the all-atom representation allows a more exact modeling of the energetics of protein-protein interfaces, it also leads to a rugged energy landscape that is hard to sample [21]. As a result, the high-resolution stage generally serves only to discriminate conformations, not to generate them.

The initial (low-resolution) structural exploration stage of common docking programs such as Haddock [20], Attract [17], ICM-DISCO [22] or RosettaDock [19] is driven by a shotgun approach of short energy minimizations started from many thousands of randomly generated initial conformations. Another large class of programs such as DOT [23], ZDOCK [18], 3D-DOCK [24], and Gramm-X [25] employ grid-based fast Fourier transform (FFT) search of rigid-body degrees of freedom to find the low-energy conformations [12], [14]. Recently, geometric hashing has been applied to quickly identify possible binding modes [26].

The philosophy behind the low-resolution sampling in most docking programs, and in particular those that employ shotgun sampling, is to guarantee an even sampling in the low-resolution stage. A contrasting philosophy is Importance Sampling, which is constructed to spend more computer time in regions of low energy than in those with high energy. It is often argued that using Importance Sampling, too much computer time is spent in a small number of low-energy regions of a potentially misleading low-resolution energy function, while a thorough exploration of conformational space is neglected. Temperature Replica Exchange, however, might overcome the lack of exploration. Thus we address the following questions in this study: First, does Importance Sampling in the form of Replica Exchange have a benefit over shotgun sampling for the low-resolution stage of protein-protein docking despite the potentially misleading low-energy function. Second, whether the highly skewed populations of conformations generated by Importance Sampling are advantageous or disadvantageous for the subsequent refinement stage.

We thus introduced *ReplicaDock*, a replica exchange Metropolis-Monte Carlo method [27], [28] for the low-resolution stage of protein-protein docking, which has been implemented within the RosettaDock program. We chose temperature levels such that the lowest temperature reflects a bound state and the highest temperature an unbound state within the RosettaDock *centroid* energy function. Within the unbound state, the binding partners are free to sample the whole surface. In the bound state, the binding partners stick together and explore local conformational space to find the lowest energy conformation accessible within the current binding mode [29].

Of course protein-binding partners would freely diffuse in the unbound state, rendering collision events rather rare. To counter-act this physical but undesired behavior we introduced an artificial restraint energy. This *encounter constraint* is a flat-bottom restraint energy that acts on the distance of the center of mass of both binding partners. As it penalizes only those conformations that are too far away to touch, the encounter constraint has no effect on the bound conformational ensemble whatsoever. However, by constricting the available conformational space volume, it increases the local concentration of the binding partners and enhances their collision rate dramatically.

As prediction of protein complex structures is still an unsolved problem [30], protein-protein docking programs are most useful in combination with sparse experimental data [6], [20]. Nevertheless, here, we tested ReplicaDock without any additional experimental data to fully focus on the sampling strategy and exclude any other possible interference factors. Performance is compared to shotgun sampling, and enumerative sampling (represented by ZDOCK) foremost via the ability to sample the lowest-energy structures after refinement and secondly by the ability to sample near-native conformations. Furthermore, we analyzed how well accurate predictions of native conformations are possible after all-atom refinement with Rosetta.

The manuscript is organized as follows. At first, we analyze the shotgun sampling employed currently in RosettaDock [19] and demonstrate that it strongly depends on the initial random placement and little on the energy function (Section 4.1). Subsequently, ReplicaDock sampling is introduced and it is showcased at hand of target 1ppf (Section 4.2). In Section 4.3, we show that near-native sampling below 4 Å I_rms to the native conformation is a necessary condition to achieve a positive effect on structural accuracy in subsequent refinement. Thus, we analyze the frequency of “hits” over a benchmark of 30 proteins for shotgun and ReplicaDock sampling (Section 4.4), and compare to ZDOCK (Section 4.5). Next, we consider all-atom refinement and analyze the sampling of distinct energy landscape features and the recovery of the native energy basins (Section 4.6). Finally, we analyze the capability to predict accurate complex structures from the refined shotgun, ReplicaDock and ZDOCK ensembles, respectively, by employing commonly employed metrics of prediction quality (Section 4.7). As discussed above, Importance Sampling might suffer from a misleading low-resolution energy function, and indeed we find some targets in the benchmark where this is the case. We discuss some of the shortcomings of the low-resolution energy function of RosettaDock that lead to alternative non-native binding modes in Section 4.8.

## Methods

### 3.1 Energy Function

#### 3.1.1 Low-resolution energy

The low-resolution stage uses the *interchain_cen* energy function which has been previously introduced for RosettaDock [19], [31]. This energy function consists of a term to reward contacting residues (*interchain_contact*), a penalty term for overlapping residues (*interchain_vdw*), a docking-specific statistical residue environment (*interchain_env*) and residue-residue pair-wise potentials (*interchain_pair*) with weights 2.0, 1.0, 1.0 and 1.0, respectively.

The *interchain_contact* component of the low-resolution energy function in Rosetta has originally been capped at −10 Rosetta Energy Units (REU) [19], which corresponds to 40 contacting residues within a distance cutoff of 6 Å between centroid-interaction centers of the two binding partners. The centroid pseudo atom is the interaction center representing all sidechain atoms in Rosetta's low-resolution representation. The cap avoids over-stabilization of spurious binding interfaces with large contact area, but also has the disadvantage that perturbations away from an optimal conformation with a large contact area are no longer penalized. Here, we obtained optimal performance by combining conformations sampled with and without energy capping. Thus, if not otherwise noted, for Shotgun (Section 3.3) and ReplicaDock (Section 3.4) always half of the generated conformations in low-resolution stage are sampled with capping at −10 REU. For ReplicaDock this is realized by running 2 of 4 trajectories with the capped energy function.

#### 3.1.2 All-atom energy

The high-resolution stage uses the standard all-atom energy for RosettaDock as given by the weight-set *docking* [31]. For final analysis an interface energy is computed by subtracting the all-atom energy of non-interacting partners from the all-atom energy of the interacting binding partners. To compute the energy of non-interacting partners the two binding partners are moved far away from each other while keeping all internal degrees of freedom fixed.

### 3.2 Generating initial conformations

To generate initial conformations we randomly perturb the orientation of both binding partners. To this end, we uniformly draw rotation matrices from the rotation group *SO*(3) by generating Euler angles *α*, *β*, *γ* (in *z*, *y*, *z* notation) with α, *γ* drawn uniformly from the interval 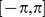 and setting 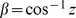 with *z* drawn uniformly from interval 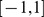


Subsequently, the binding partners are slid into contact using steps of 1 Å by first increasing the distance between the binding partners until the energy term *interchain_vdw <0.1*, and then again decreasing until *interchain_vdw >0.1*.

### 3.3 Shotgun Protocol (low-resolution stage)

Shotgun sampling in RosettaDock's low-resolution stage proceeds in two steps. First, a random initial conformation is generated as described in the preceding section. Second, a Monte-Carlo (MC) sampling procedure with 500 steps is applied to optimize the low-resolution energy. At step *i* a proposed conformation 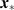 is accepted according to the Metropolis Criterion 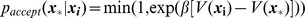 where 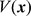 denotes the potential energy of conformation 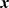, and 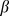 the inverse temperature. The inverse temperature is kept constant at 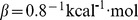 and the step-sizes are adjusted every 50 steps to maintain a 50% acceptance rate. The initial step-sizes are drawn from normal distributions with mean value of 0.7 Å (translation along all the three axes) and 5° (rotation around the axis of protein centers and tilt off this axis in a randomly-chosen direction) [19]. At the end, the lowest energy conformation observed during the 500 MC-steps is recorded as final output.

By presetting the number of generated decoys (*-nstruct*) we adjusted the computer time expense to match ReplicaDock's expense. We have generated about 120,000 decoys with shotgun sampling for each target. Decoys with *interchain_contact* >10 are discarded and the top 40,000 in energy are selected for analysis or refinement. (Method S1: protocol_capture/rosetta_dock/centroid in Supporting Information S1)

### 3.4 ReplicaDock (alternative low-resolution stage)

As an alternative to the shotgun sampling described above we applied here a replica exchange procedure [27], [28]. Inverse temperatures are set to, *β*, of 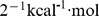, 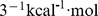
and 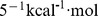, and swaps are attempted every 1,000 Monte-Carlo steps [32], [33]. 4 trajectories with 3 temperature levels are run for 5×10^6^ Monte-Carlo steps, and snapshots are stored every 1,000 steps. In total, 60,000 decoys are generated for each target with this protocol. For further analysis or refinement, the highest temperature level (inverse temperature 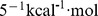) and decoys with *interchain_cen* >10 were excluded. Initial configurations of trajectories were generated as described in Section 3.2. All targets of the benchmark are sampled with the same three temperature levels. The choice of temperature levels is discussed in Results Section 4.2. For all targets good exchange rates (∼25%) are achieved and no further target dependent optimization is required.

To avoid unbounded diffusion of the two binding partners away from each other, we generated an *encounter constraint*. This constraint is realized as flat-bottom distance restraint between the 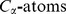 closest to the center of mass of the respective binding partners. These center atoms are denoted in the following as 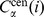 with 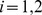. The constraint does not penalize the conformations unless they are further than 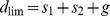 apart, where *g* denotes the chosen gap parameter (here 8 Å), and 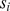 denotes the furthest distance of a surface 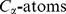 of binding partner *i* to its center 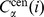. For distances 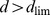 the harmonic penalty energy 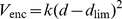 is applied. (Method S2: protocol_capture/replica_dock/centroid in Supporting Information S1)

### 3.5 ZDOCK

To prepare for ZDOCK, protons are removed and the individual binding partners are marked using *mark_sur* from ZDOCK's toolbox. 54,000 decoys are generated with ZDOCK3.0.2 for each target and top 36,000 decoys by ZDOCK score are evaluated and refined (Section 3.6).

### 3.6 Refinement (high-resolution stage)

The high-resolution stage of RosettaDock described by *Gray et al.* [19] was applied without alteration and used to refine conformations generated with shotgun, ZDOCK or ReplicaDock. (Method S3: protocol_capture/rosetta_dock/refine and Method S4: protocol_capture/replica_dock/refine in Supporting Information S1)

### 3.7 Construction of Benchmark

30 Targets were selected from the Dockground Benchmark [34], for which the x-ray resolution of the bound complex is no worse than 2.5 Å and the x-ray resolution of the individual unbound partner is no worse than 2.2 Å. Since the current method does not allow backbone motion, we restricted the benchmark to targets where the monomer 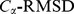 between bound and unbound structure is 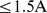 for both binding partners (Table S1).

### 3.8 Implementation of ReplicaDock in Rosetta3

We implemented replica exchange within the general Metropolis-Hastings framework of the Rosetta3 software package. The replica-exchange module is accessible through the *RosettaScripts* [35] interface and can be combined with any conformational moves that are implemented as children of the *ThermodynamicMover* class. To ensure detailed balance, either Movers have to yield unbiased conformational perturbations or they have to provide the proposal density of the perturbation through implementation of an abstract virtual function in the *ThermodynamicMover* interface. For docking we have provided the *ThermodynamicRigidBodyPerturbNoCenterMover* which performs unbiased rotational and translational moves. Random translations drawn from Gaussian distribution are performed along all three axes. The axis-angle notation is used to represent rotations. A rotation axis is generated using
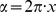


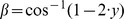


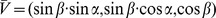
where *x*, *y* are randomly drawn from uniform distribution. This is sufficient to guarantee unbiased rotational sampling, and a distribution for the positive rotation angle can be chosen freely. In order to be consistent in rotational step sizes with the parameterization of the original RigidBodyPerturbNoCenterMover, we first draw Euler angles from a Gaussian distribution with specified magnitude, and then transform the resulting rotation into axis-angle representation. Second, we combine the rotation axis obtained using our unbiased sampling method, with the rotation angle that corresponds to the Gaussian Euler angles. The resulting distribution of rotation angles is shown for different parameters in Figure S1 in Supporting Information S1.

Additionally, we have implemented the *DockSetupMover* to select the rigid-body degrees of freedom to be sampled (via *FoldTree*) and implemented the *parse_my_tag* method of the existing *DockingInitialPerturbationMover* [31] to render it accessible through the RosettaScripts interface.

### 3.9 Metrics for structural accuracy and docking performance

The metrics interface RMSD (I_rms), ligand RMSD (L_rms), fraction of native contacts (*f_nat_*) and fraction of non-native contacts (*f_non-nat_*) are defined as in CAPRI [36] and are calculated against the bound complex.

For the low-resolution ensemble we consider a decoy with I_rms 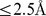 as ‘hit’.

### 3.10 Sampling the native energy basin

Additionally, we were interested in the native energy basin accessible by the applied fixed-backbone, flexible-sidechain docking protocol from the unbound monomeric starting structures. Accordingly, we started 1000 trajectories of refinement (Section 3.6) from the unbound monomers superimposed onto the bound complex to generate *RelaxedNative* ensembles. (Method S5: protocol_capture/relax_native in Supporting Information S1)

To assess how well a protocol samples the native energy basin, we count conformations that overlap with the RelaxedNative ensembles as follows. A lower left region in the interface energy vs. I_rms plots relative to the RelaxedNative ensemble was defined by the 50%-tile interface energy of RelaxedNatives as upper confinement and by the 75%-tile I_rms of the RelaxedNatives as the right confinement (Figure S2 in Supporting Information S1). All conformations in this region are counted.

We define 4 categories based on the number of conformations *n* that overlap with the RelaxedNative ensemble: none (0), magic points (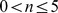), sporadic (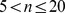), dense (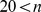).

### 3.11 Clustering after all-atom refinement

The top-2000 conformations by interface energy after all-atom refinement were clustered using a cutoff of 5.0 Å of 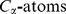 RMSD. Clusters are ranked by size and are represented by the decoy with lowest interface energy within the cluster.

Ranking clusters by interface energy resulted in slightly worse performance when evaluated using CAPRI criteria (Table S2).

### 3.12 Automated Setup

The automated setup tools available with the CS-Rosetta toolbox (www.csrosetta.org) have been used to generate all production runs of the benchmark. We advise users to install this toolbox from *protocol_capture/2012/replica_docking/csrosetta3* or from the website. If installed from the website, the docking plugins *_docking_base*, *rosetta_dock* and *replica_dock* have to be copied into the *flag_library/methods* folder from*/protocol_capture/2012/replica_docking/csrosetta3/flag_library/methods*. All methods presented in this work are implemented as plugins in *csrosetta3/flag_library* and can be accessed through the *–method* option of the *setup_xxx* commands. Example usage of these tools for docking can be found in the protocol capture section (Figure S3 in Supporting Information S1, Method S7: Automated Setup in Supporting Information S1) and general documentation is provided at www.csrosetta.org/manual.

### 3.13 Computational Cost

For shotgun sampling and ReplicaDock the same amount of computer time was used. The amount of computer time required depends on the size of the binding partners and ranges from 66 core-hours to 900 core-hours on 2.6 GHz AMD Opteron Processors. ZDOCK requires significantly less computer time, with 1.25–7.25 core-hours on the same machine. We refined 40,000, 36,000 and ∼36,000 conformations for shotgun, ZDOCK and ReplicaDock, respectively. Refinement requires between 14–120 core-seconds per conformation, again depending strongly on the protein sizes.

## Results

### 4.1 Shotgun sampling is dominated by initial random placement

In this section we address the question how conformational space is explored in the shotgun approach of RosettaDock. Shotgun sampling consists of a random initial placement followed by a short energy optimization procedure using 500 steps of Monte Carlo sampling with adjusted step-sizes and constant inverse temperature of 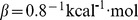
(Methods).

**Figure 1:**
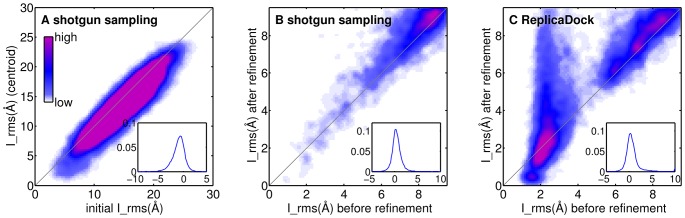
Detailed analysis of individual docking stages on bound target 1sq2. A) Interface RMSD (I_rms) before and after the Monte-Carlo optimization in the low-resolution stage of RosettaDock's shotgun sampling, B–C) I_rms before and after all-atom refinement for shotgun and ReplicaDock sampled ensembles, respectively. The colorbar indicates the density of data points at given position of the scatter plot, A–C) use a same colorbar range. The insets show the distribution of differences between I_rms after and before the respective sampling stage has been applied (negative values reflect an improvement in I_rms).

**Figure 2:**
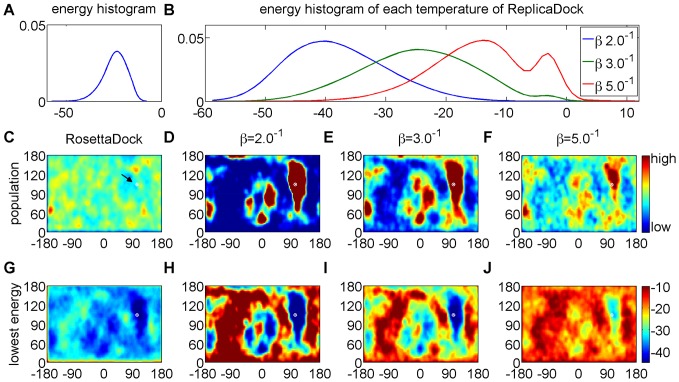
Detailed analysis of shotgun and ReplicaDock sampling on target 1ppf. A) energy distribution of shotgun sampling generated low-resolution decoys. B) energy distribution of conformations sampled by ReplicaDock at respective inverse temperatures. C–F) Population of sampled conformations in spherical coordinates. Partner A is fixed at the center and the position of Partner B with respect to an idealized spherical surface around Partner A is recorded. The native structure is labeled as white dot (arrow in C). G–J) Conformations are assigned to grid-cells as in C–F, but shown is the lowest energy of all conformations assigned to the respective grid cell. The same color-scale is used for each plot of a row, and the colorbars are attached to the rightmost panel.

The short Monte-Carlo sampling explores only a small region of conformational space around the respective starting position, which is reflected by a strong correlation between I_rms to the native complex structure before and after the Monte Carlo optimization (Figure 1A). Thus, the generated ensemble of structures is highly biased by the initial starting structures, rather than by the low-resolution energy (Figure 2C+G; Figure S4C+G in Supporting Information S1).

Subsequently, the decoys of the low-resolution phase are refined using an all-atom model and allowing side-chain flexibility on top of the rigid body motion. The energy landscape in this phase is very rugged and the applied refinement protocol is not very explorative. Indeed, the I_rms of low-resolution input structures and all-atom refined structures are also strongly correlated (Figure 1B). Thus, a necessary requirement for the prediction of the native complex structure is that the initial random placement already generates sufficiently many near-native conformations.

### 4.2 Sampling with ReplicaDock generates energy-biased populations

The previous section showed that the relative populations of conformations in the shotgun ensemble are dominated by the initial perturbation and do not reflect the low-resolution energy landscape. Nevertheless, differences in energy are apparent and the native structure is found in one of the low-energy basins (Figure 2G).

We argued that Importance Sampling might generate ensembles of better quality with more near-native conformations that are better suited for continuation in the high-resolution refinement stage. Accordingly, we employ unbiased rigid body moves and replica exchange Monte-Carlo (REXMC). The temperature levels are chosen such that the lowest and highest temperatures reflect the bound and unbound state, respectively.

To achieve efficient exchange rates between replica's, the energy distribution at any given temperature level has to overlap with the energy distributions of neighboring temperature levels. Generally, in applications of replica exchange to biomolecular systems this can only be achieved by employing a large number of temperature levels combined with careful optimization for each individual system [37]–[39]. Here, however, only the six rigid body degrees of freedom are sampled rendering energy distributions rather broad, such that only a small number of temperature levels is required [40]. In an initial survey, simulations on bound target 1sq2 were carried out with inverse temperatures, *β*, 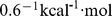, 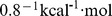, 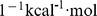, 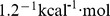, 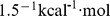, 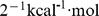, 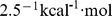, 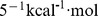 and 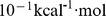 (Section 3.3, Figure S5 in Supporting Information S1). We found that the lowest temperature levels lead to sharp energy distributions centered at relatively low energies. Inspection of the corresponding conformations reveals predominantly large number of contacts consistent with bound conformations. Another peak in the energy distribution is found at the higher temperature levels, and inspection of the corresponding conformations shows that binding partners have little or no contact. Inverse temperatures between 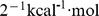 and 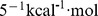 yield broad distributions that connect the low-energies of bound conformations with the sharp energy-peak of unbound conformations. In our initial tests, this temperature range turned out to be most suitable to broadly sample different binding modes, and we chose the inverse temperatures (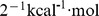, 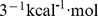 and 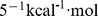) for the final protocol. This choice yields consistent results across the full range of benchmark cases in terms of energy distributions (Figure S6 in Supporting Information S1) and exchange rates around 25% between replica's with little variation between targets (Table S3).

Replica Exchange with inverse temperatures 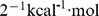, 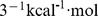 and 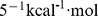 is show-cased at the example of target 1ppf in Figure 2. Indeed, ReplicaDock achieves good overlap in the energy distributions (Figure 2B) and frequent exchanges (Figure S7 in Supporting Information S1). Obviously, at high temperature the unbound (non-contacting) conformations are more populated, whereas at low temperatures bound (contacting) conformations are more populated. Thus, thermodynamically speaking, the high-temperature state reflects the unbound state, whereas the low-temperature state reflects the bound state, and the replica exchange scheme achieves frequent exchange between both.

Independent trajectories started from different random conformations converge to the same populations (Figure S8 in Supporting Information S1) demonstrating that convergence is achieved within the simulation time. Accordingly, all generated trajectories can be combined to improve statistics (Figure 2D–F). As expected from Importance Sampling, areas of high population in the ReplicaDock ensemble coincide with the low-energy regions in the conformational energy landscape (Figure 2H–J), which is in stark contrast to the shotgun ensemble.

Surprisingly, with the lowest temperature set to 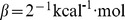, ReplicaDock achieved significantly lower energies than shotgun sampling with temperatures set to 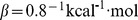. This demonstrates that significantly more than 500 Monte-Carlo steps, as employed in the shotgun protocol, are required to consistently equilibrate at the chosen temperature.

### 4.3 Only near-native conformations are pulled into the native energy funnel in the refinement stage

Refinement of the ReplicaDock ensemble results in a dramatically different behavior than refinement of the corresponding shotgun ensemble (Figure 1B+C). When conformations below I_rms 3 Å are refined, they are likely to be pulled into the native energy funnel, resulting in I_rms between 0 and 1 Å (Figure 1B+C). Decoys further than 4 Å do not systematically improve their I_rms in refinement.

Since the shotgun centroid ensemble has a very low number of conformations with I_rms 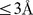 refinement is less beneficial for shotgun than for ReplicaDock ensembles. However, for ReplicaDock decoys, some refinement trajectories also seem to move away from the near-native regions. This seems to be caused by clashes after switching to the high-resolution representation (data not shown). As discussed later (Section 4.8), the centroid energy is dominated by *interchain_cen* which may cause some overly compact conformations.

### 4.4 ReplicaDock efficiently samples near native conformations

In the previous section, we showed dense sampling of near native conformations during the low-resolution phase is a necessary condition for identifying the native energy basin in the refinement phase. In the following, we analyze how successful the shotgun approach and ReplicaDock sample near-native conformations in the low-resolution phase on a benchmark of 30 protein-protein complexes (Methods), where the docking partners are given as PDB-structures of the unbound proteins.

We applied ReplicaDock with inverse temperatures, *β*, 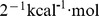, 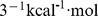 and 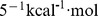
for low-resolution sampling. For ReplicaDock, 4 trajectories with 5×10^6^ MC-steps were run using different amounts of computer time depending on the target size. The same amount of computer time per target was given to shotgun sampling by adjusting the number of generated decoys (*-nstruct*), accordingly.

**Figure 3:**
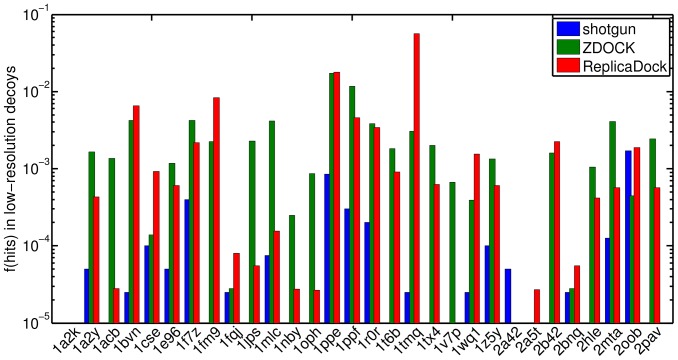
Fraction of hits in low-resolution docking. Conformation with I_rms ≤2.5 Å to the bound complex are considered as a hit (Section 3.9). Blue, green and red represent the results of shotgun sampling, ZDOCK and ReplicaDock, respectively.

As shown in Figure 3, ReplicaDock generates significantly higher fractions of near-native decoys for nearly all targets than shotgun sampling. In three cases (1a2k, 1v7p, and 2a42) ReplicaDock did not generate any near-native decoys. Scrutinizing the targets in detail, we find that for 1a2k a few residues on the terminus of the receptor stick into the binding pocket and cause clashes between backbone atoms in the superimposed structures. Similarly, in complex structure 1v7p and 2a42 loops in the interface area deviate substantially from the position they occupy in the unbound monomer conformation. Without these backbone changes crucial side-chains cannot be packed without clashes in the interface, causing the binding partners being pushed apart. In these cases shotgun sampling also produced only a small fraction of near-native decoys. Obviously, these problems cannot be overcome with rigid-body docking, and thus explain the bad performance of the methods.

Additionally, if we impose further energy cut-offs and only keep the low-20k, low-5k or low-2k of conformations (Figure S9 in Supporting Information S1), we find that a) for an increasing number of targets no near-native conformations are retained, and b) that the fraction of near-native decoys become more similar between shotgun and ReplicaDock sampling. This shows that ranking by low-resolution energy is problematic and only generous filters should be applied before refinement.

In summary, ReplicaDock yields a dramatic improvement of near-native sampling for most targets. Some targets, however, are not improved or get slightly worse, and aggressive energy-cuts have a negative impact. Possible reason for these failures of the low-resolution energy function will be discussed further in Section 4.8.

### 4.5 Comparison to ZDOCK

As shown above, ReplicaDock yields ensembles with a higher fraction of near-native decoys than RosettaDock's shotgun sampling method. Another large class of docking programs uses enumerative sampling on a translational and rotational grid. Here, we run ZDOCK 3.0 as a representative of these programs. ZDOCK has been shown to be one of the most successful docking programs [41]–[43].

As shown in Figure 3, the performance of ZDOCK and ReplicaDock is comparable. For most targets both methods yield fractions of near-native decoys between 10^−4^ and 10^−2^. Moreover, both methods fail in some targets: 2a42 and 2a5t for ZDOCK, and 1a2k, 1v7p and 2a42 for ReplicaDock. However, one should mention that ZDOCK is more efficient due to its FFT based sampling of translational degrees of freedom and requires only a small fraction (∼1%) of the computational expense (Methods).

### 4.6 All-atom refinement of ReplicaDock ensembles

As shown above, ReplicaDock and ZDOCK yield ensembles with a higher fraction of near-native decoys than shotgun sampling. To analyze whether these improvements also have a positive impact on the all-atom refinement stage of the docking protocol, we refined the shotgun, ZDOCK and ReplicaDock ensembles for all targets in the benchmark. For ReplicaDock and shotgun sampling we have made sure that ReplicaDock use always equal or slightly less overall computer time compared to the shotgun based approach. In any case the computational expense for refinement of any conformation, whether generated by ZDOCK, ReplicaDock or shotgun sampling is approximately the same.

Next we want to test the respective sampling strategies for their ability to detect the native energy basin of the all-atom energy function after refinement. To show the position and form of the native energy funnel, we also generated RelaxedNative ensembles (Methods) by starting multiple trajectories of refinement from unbound monomers superimposed onto the bound complex.

**Figure 4:**
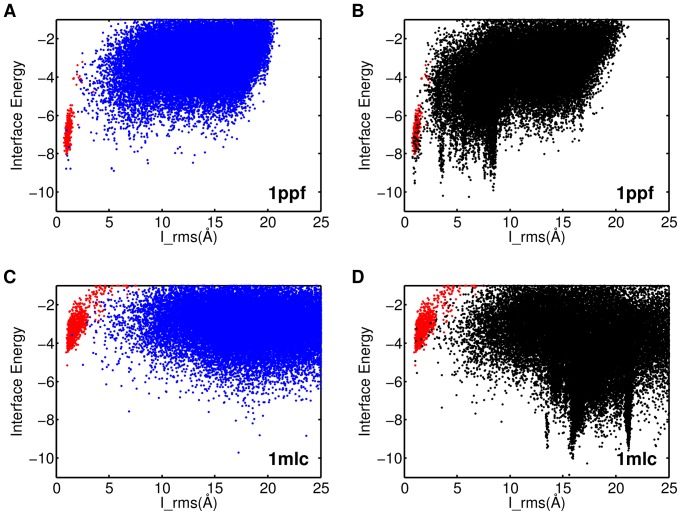
Interface RMSD vs. Interface Energy after refinement on target 1ppf and 1mlc. A) and C) refinement of shotgun sampling generated ensembles, B) and D) refinement of ReplicaDock generated ensembles. The red dots represent the RelaxedNative ensembles (Results).

We first introduce the typical differences between refined shotgun and ReplicaDock ensembles at the example of target 1ppf and 1mlc. As shown in Figure 4A, the refined shotgun ensemble of target 1ppf (blue) is higher in energy than the RelaxedNative ensemble (red). Only 3–4 isolated conformations reach significantly lower energies. The ReplicaDock ensemble, on the contrary, shows three distinct energy funnels that are well sampled (Figure 4B). One of the funnels coincides in form and position with the funnel formed by the RelaxedNative structures, demonstrating that the native energy basin has been found and is well sampled. But unfortunately it is neither the lowest nor the most pronounced energy funnel, rendering discrimination of native from non-native decoys challenging. Apparently, the Rosetta all-atom energy function features at least three well-resolved energy funnels for this target complex, which is confirmed by finding corresponding clusters that are well populated (data not shown). All three of these funnels remain poorly sampled with shotgun sampling. For target 1mlc the RelaxedNative conformations have higher energies than the shotgun or ReplicaDock ensembles (Figure 4C+D). This points to deficiencies of the energy function. However, also for 1mlc shotgun sampling produces only sporadic sampling of low energies, whereas ReplicaDock detects distinct funnels in the energy landscape.

**Figure 5:**
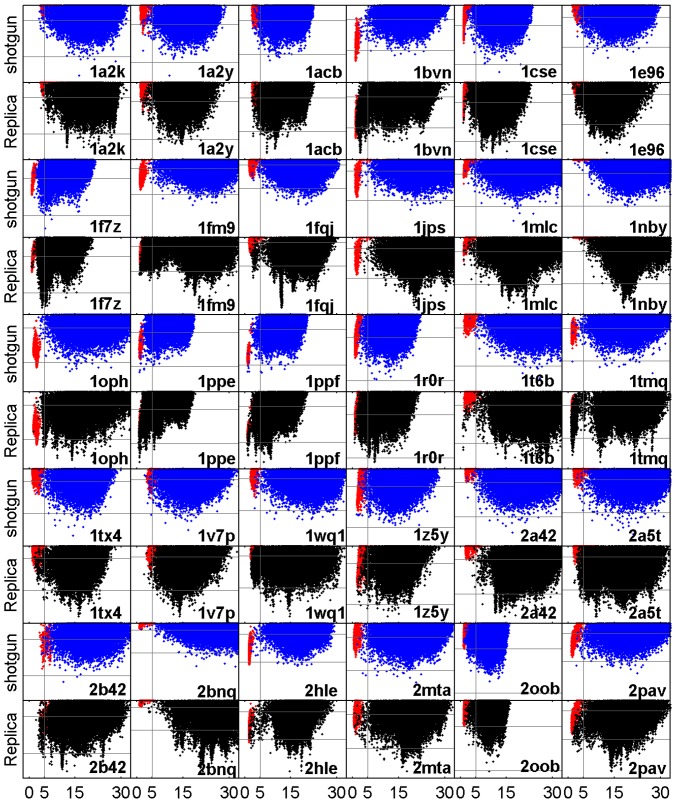
Interface RMSD vs. Interface Energy after refinement for all the 30 unbound docking targets. The red dots represent the RelaxedNative ensembles(Results). The interface RMSD is shown on the *x*-axis, the interface energy on the *y*-axis. The same energy range is used for displaying both, shotgun sampling (blue) and ReplicaDock (black), results of each target, respectively. The vertical gray lines correspond to I_rms of 5.0 Å, and the two horizontal gray lines correspond to interface energy −4 and −8 Rosetta Energy Units.

**Figure 6:**
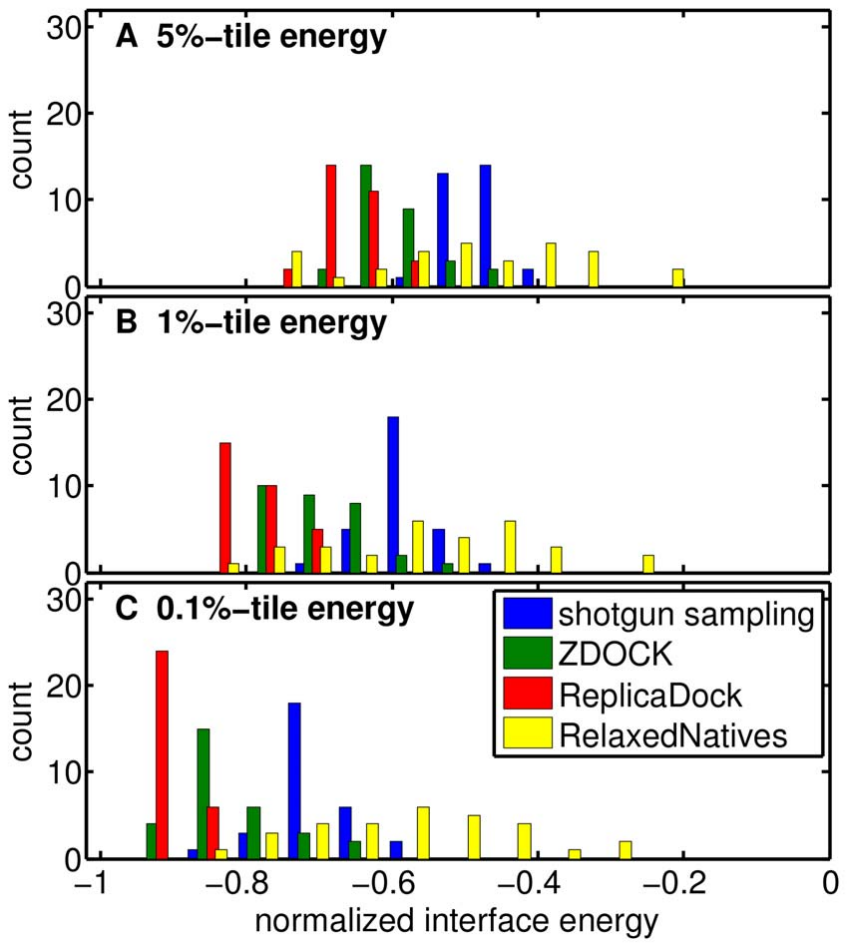
Interface energy distribution obtained with different simulation protocols. The energies for each target and method have been normallized to the dynamic range of interface energies observed for the respective target across all methods. The interface energies are normalized by the absolute value of mean energy of the 10 lowest observed energies for this target (highest energy is always 0). The *x*-percentile energy is the scaled energy value that separates off *x*% of the lowest energy decoys for a given simulation result. Shown are the distributions of *x*-percentile energies across all 30 targets for a) the 5%-tile, b) the 1%-tile and c) the 0.1%-tile, respectively.

The main observations for ReplicaDock for targets 1ppf and 1mlc are a) that much lower energies are sampled, b) that distinct energy funnels are sampled densely, and c) that for 1ppf the native energy funnel is sampled densely. Next, we ask whether similar differences in behavior between shotgun and ReplicaDock are observable for all 30 targets. Indeed, equivalent scatter plots of all targets (Figure 5) show similar differences between shotgun and ReplicaDock as already observed for targets 1ppf and 1mlc. To quantify, we computed histograms of the lowest energies sampled per target by the respective approaches (shotgun, ZDOCK, ReplicaDock and RelaxedNative). Whether we focus on the lowest 0.1%, 1% or 5% of decoys, energies of shotgun ensembles are higher for all targets, and even the RelaxedNative ensembles often do not reach energies as low as ReplicaDock (Figure 6). Energies of refined ZDOCK conformations are in-between those of ReplicaDock and shotgun. These results demonstrate that the conformations in the centroid ReplicaDock ensemble are well poised to reach low interface energies in the subsequent all-atom refinement.

For target 1ppf and 1mlc, ReplicaDock detected funnels in the energy landscape that were not revealed by shotgun ensembles. The same impression is reached from a comparison of the sampling methods on all targets (Figure 5). Refined ZDOCK ensembles are more comparable with refined ReplicaDock ensembles than shotgun ensembles, but also show slightly less distinct energy funnels overall (Figure S10 in Supporting Information S1).

We would like to quantify the performance of the two sampling strategies in detecting funnels in the energy landscape. However, *a priori* it is impossible to tell how many and which funnels should be detected by an optimal sampling strategy. The only funnel whose existence is known *a priori* is the funnel formed by the RelaxedNative ensemble (red). We thus restrict ourselves to ask whether overlap with the RelaxedNative ensemble is achieved. Model selection is often based on clustering, and it has been shown that cluster size is an important criterion for ranking that it is often found to be more reliable than energies [44]. Thus a relatively high population is crucial for the confident selection of a binding mode. Accordingly, we discriminate four cases (Methods): a) *dense*, the native funnel is densely sampled (1ppf; ReplicaDock) b) *sporadic*, the native funnel is sampled sporadically (1ppe; shotgun or 1a2y; ReplicaDock) c) *magic points*, individual decoy structures coincide with the native funnel (1fqj; shotgun) and d) *none*, no decoy structures fall within the native funnel (2hle; shotgun).

The individual classifications for shotgun, ZDOCK and ReplicaDock are shown in Table 1. In summary, we found 2 *densely* sampled native funnels for shotgun but 22 and 19 for ZDOCK and ReplicaDock, respectively. *No funnel* is found for 8 targets in shotgun ensembles, and for 2 targets in ZDOCK or ReplicaDock ensembles. For 13 targets, ReplicaDock improves the category by at least two steps, and for 11 targets by one step. ReplicaDock and ZDOCK dramatically improves the ability to detect existing funnels in the energy landscape compared to the shotgun approach.

**Table 1:** Qualitative classification in ability to sample native energy basin.

target	shotgun	ZDOCK	ReplicaDock
1a2k	dense	magic	dense
1a2y	magic	dense	sporadic
1acb	magic	dense	dense
1bvn	none	dense	dense
1cse	magic	magic	dense
1e96	sporadic	dense	dense
1f7z	none	dense	sporadic
1fm9	none	dense	dense
1fqj	magic	magic	sporadic
1jps	none	sporadic	none
1mlc	magic	dense	sporadic
1nby	sporadic	dense	dense
1oph	none	dense	magic
1ppe	sporadic	dense	dense
1ppf	magic	dense	dense
1r0r	magic	dense	dense
1t6b	magic	dense	dense
1tmq	magic	dense	dense
1tx4	magic	dense	dense
1v7p	dense	dense	dense
1wq1	magic	dense	dense
1z5y	sporadic	dense	dense
2a42	magic	none	none
2a5t	sporadic	dense	dense
2b42	sporadic	dense	dense
2bnq	magic	none	magic
2hle	none	sporadic	sporadic
2mta	none	dense	magic
2oob	sporadic	magic	magic
2pav	none	dense	dense
dense	2	22	19
sporadic	7	2	5
magic	13	4	4
none	8	2	2

At the end is the summary of each category achieved by each method.

### 4.7 Performance in structure prediction/ranking

In previous sections we established that ZDOCK and ReplicaDock dramatically improve sampling in the initial docking stage compared to shotgun sampling, and refined ensembles of ReplicaDock reveal many more distinct funnel like features in the high-resolution energy landscape. However, we also found that the improved probing of the high-resolution energy landscape reveals many non-native energy funnels with lower energies than the native energy. Thus, it is not clear whether the improved sampling also leads to improved structure prediction in RosettaDock for unbound docking with relatively rigid targets as they were selected for our benchmark (Methods).

To address this question, we clustered refined ensembles and ranked clusters by size. From the ten largest clusters we selected the lowest interface energy model and evaluated using CAPRI criteria. As customary in CAPRI assessment we report metrics for the best of the ten models (Table 2). As a summary, ZDOCK and ReplicaDock performed both better than shotgun in structure prediction, but ZDOCK displayed the overall best performance. In CAPRI, 0, 1, 2 or 3 stars are awarded for incorrect, acceptable, medium and high quality models [36]. Here, ZDOCK, ReplicaDock and shotgun generate acceptable or better models for 14, 8 and 5 targets, respectively. Indeed, the consistent appearance of non-native energy funnels in ReplicaDock ensembles obviously impedes concise selection of the native funnel despite its dense sampling.

**Table 2:** Summary of structure prediction accuracy in unbound docking.

	shotgun refined					ZDOCK refined					ReplicaDock refined				
Target	CQ^1^	L_rms	I_rms	*f_nat_*	*f_nonnat_*	CQ^1^	L_rms	I_rms	*f_nat_*	*f_nonnat_*	CQ^1^	L_rms	I_rms	*f_nat_*	*f_nonnat_*
1a2k	0	43.76	13.51	0.000	1.000	0	38.93	15.42	0.000	1.062	0	30.01	12.32	0.146	1.729
1a2y	0	21.73	12.39	0.000	1.295	0	22.91	6.98	0.068	1.455	0	24.48	7.64	0.023	1.273
1acb	0	17.51	7.62	0.014	0.667	*	9.81	3.89	0.145	0.899	**	4.87	2.70	0.333	0.580
1bvn	0	17.78	7.68	0.068	0.753	0	14.73	6.10	0.110	1.014	*	4.72	2.48	0.274	0.781
1cse	0	17.63	7.98	0.013	1.228	0	18.93	7.81	0.000	0.823	*	12.44	3.90	0.241	0.544
1e96	0	39.15	8.17	0.000	1.366	0	17.80	6.26	0.122	1.146	0	27.70	7.01	0.000	1.951
1f7z	0	17.72	3.81	0.098	0.705	0	14.40	3.73	0.066	1.066	0	17.70	4.16	0.197	0.836
1fm9	0	49.56	17.72	0.000	0.606	**	4.03	2.60	0.364	0.455	0	58.34	19.29	0.000	1.242
1fqj	0	40.84	12.25	0.000	1.283	0	43.10	13.27	0.000	1.150	0	27.66	11.89	0.000	2.250
1jps	0	28.48	7.35	0.014	0.803	*	13.23	3.17	0.254	0.620	0	25.41	10.73	0.000	1.070
1mlc	0	56.02	15.87	0.000	1.036	0	26.80	6.88	0.054	0.964	0	56.51	12.38	0.000	1.250
1nby	0	45.97	13.09	0.000	1.000	0	19.89	10.58	0.041	0.743	0	52.93	14.69	0.000	1.135
1oph	0	50.10	17.46	0.000	0.806	**	5.07	2.56	0.581	0.306	0	19.40	4.60	0.145	0.935
1ppe	***	2.65	0.95	0.746	0.085	***	1.76	0.79	0.761	0.197	***	2.11	0.88	0.775	0.113
1ppf	***	2.69	0.94	0.824	0.196	**	3.57	1.12	0.824	0.275	***	2.89	0.99	0.745	0.255
1r0r	0	15.11	7.13	0.106	0.621	*	10.35	2.76	0.394	0.424	*	9.17	2.47	0.455	0.394
1t6b	0	69.14	24.11	0.000	1.338	0	17.94	10.19	0.000	0.815	0	69.09	19.68	0.000	1.415
1tmq	0	19.81	11.88	0.026	0.776	**	4.94	1.43	0.579	0.447	**	4.45	1.36	0.618	0.513
1tx4	0	27.23	9.99	0.000	1.015	0	28.38	12.40	0.031	0.954	0	22.10	11.11	0.062	1.200
1v7p	0	26.58	14.58	0.016	1.081	0	23.99	8.62	0.145	1.081	0	23.53	11.85	0.048	1.290
1wq1	0	14.01	7.10	0.121	0.527	*	7.27	3.80	0.220	0.396	0	11.50	6.87	0.011	1.011
1z5y	*	4.12	1.92	0.273	0.424	*	6.18	3.22	0.333	0.500	0	10.22	5.68	0.076	0.697
2a42	0	42.29	12.10	0.000	1.250	0	82.18	23.67	0.000	1.500	0	51.95	12.84	0.021	1.667
2a5t	0	17.50	4.82	0.186	0.797	0	23.51	14.07	0.000	0.932	0	25.50	8.97	0.068	1.102
2b42	0	21.95	11.42	0.079	0.494	*	9.28	4.15	0.157	0.742	0	17.64	7.31	0.079	0.730
2bnq	0	74.85	29.64	0.000	1.182	0	57.16	16.52	0.000	1.977	0	70.08	14.78	0.000	2.341
2hle	0	24.94	12.76	0.000	0.774	*	6.63	2.91	0.464	0.405	0	36.60	10.28	0.083	0.619
2mta	*	7.25	3.34	0.457	0.630	**	10.11	2.52	0.543	0.565	0	47.15	17.22	0.000	1.543
2oob	*	8.38	3.38	0.333	0.704	0	11.03	5.51	0.111	0.852	0	11.45	6.96	0.000	0.963
2pav	0	33.95	13.71	0.035	1.158	**	3.35	1.45	0.509	0.333	*	7.65	3.00	0.421	0.877
summary	3*/2***					7*/6**/1***					4*/2**/2***				

Clusters are ranked by size and represented by the lowest interface energy decoy. In column ‘CQ’ (CAPRI Quality),

‘0’ indicates that none of the top 10 models was of accetable quality,

‘*’, ‘**’ and ‘***’ indicates that at least one of the top 10 models is of acceptable, medium or high quality, respectively (Section 4.7).

Columns ‘L_rms’, ‘I_rms’, ‘*f_nat_*’ and ‘*f_non-nat_*’ record the respective information of the best model within these top 10 models.

1 CQ refers to CAPRI quality.

### 4.8 Targets with insufficient near-native sampling

**Figure 7:**
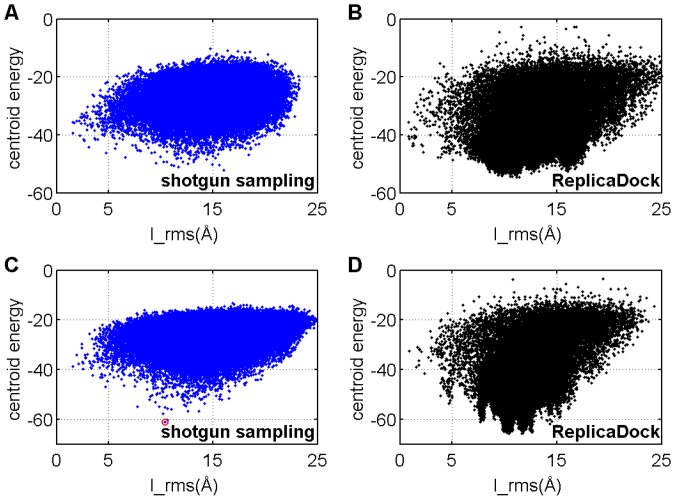
The centroid energy function prefers an alternative binding modes for the bound docking target 1emv. A–B) shotgun and ReplicaDock sample the low-resolution docking stage with ‘capped’ centroid energy function. C–D) shotgun and ReplicaDock sample the low-resolution docking stage with centroid energy function with no cap. The structure indicated by the red circle will be shown in Figure 8.

As shown in Section 4.4, for some targets none or only a few near-native decoys are sampled by both approaches. We found that in these cases the centroid energy function fails to stabilize the native conformation and/or over-stabilizes non-native conformations (Figure 7). In this section, we explore possible reasons for these failures of the low-resolution energy function.

**Figure 8:**
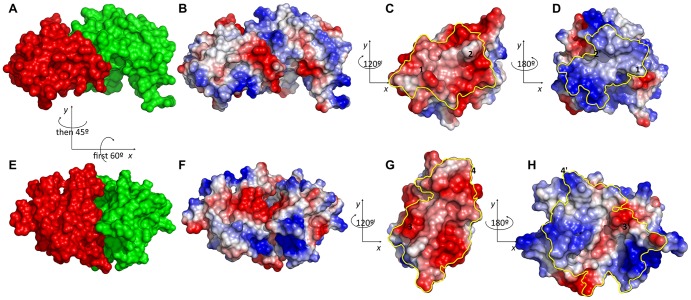
Electrostatic surface potential of native (A–D) and *interchain_cen* preferred conformation (E–H) in all-atom representation. A) native complex with receptor in green and ligand in red B) electrostatics map of the native complex C) electrostatics map of native ligand interface D) electrostatics map of native receptor interface E) *interchain_cen* preferred conformation after refinement F) electrostatics map G) electrostatics map of receptor interface H) electrostatics map of ligand interface. The yellow lines in C, D, G and H indicate the respective interface regions, and number pairs (e.g. 1 in C and 1′ in D) indicate corresponding contact regions. Relations of viewing angle are given between panels where required.

The low-resolution energies are dominated by the *interchain_contact* term, which awards large contact surfaces (Figure S11 in Supporting Information S1). Indeed, all over-stabilized alternative binding modes that we found have significantly larger contact surfaces compared to the native complex (Figure 8). For example, 1emv features a spurious binding mode with significantly larger buried surface area of 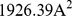 than its native state 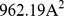 (Figure 8). Buried surface was calculated from the low-resolution models using the POPSCOMP sever [45], [46]. Performing additional docking runs starting from the bound monomers we ruled out the possibility, that lack of backbone flexibility prohibits full stabilization of the native conformation.

A qualitative comparison of the electrostatic potentials at the native and alternative binding interfaces reveals a possible energetic counter-weight to the dramatic difference in buried surface area. Whereas the native state binding mode consists of surface patches of complementary charges, the alternative binding mode would superpose surface patches of equal charge. A more quantitative analysis is necessary, but this observation suggests that the electrostatic interactions are not captured sufficiently well by the empirical *interchain_pair* and *interchain_env* potential terms.

## Conclusions

In this study, we introduced ReplicaDock, which uses temperature replica exchange to switch between bound and unbound thermodynamic states, and benchmarked its performance for sampling the low-resolution stage of protein-protein docking in RosettaDock.

For most targets tested in our benchmark, ReplicaDock reached significantly lower energies and generates drastically higher fraction of near-native decoys than shotgun sampling. After refinement, ReplicaDock-generated decoys reach lower interface energies and reveal funnel-like features of the energy landscape that are hardly visible when shotgun sampling is applied. The new funnel-like features seem to reflect distinct binding modes. Most importantly, the native energy funnels are often exactly matched in shape and position by ReplicaDock.

Enumerative search of rigid body degrees of freedom as carried out by ZDOCK 3.0 yields similar results as ReplicaDock. It also yields a higher fraction of near-native decoys and lower energies after refinement with Rosetta when compared to shotgun sampling. But ZDOCK does not reveal quite as many distinct funnels in the high-resolution Rosetta energy landscape as ReplicaDock. This is an advantage when the goal is to predict complex structures with the correct energy function. However, to improve methods further, it is important to have sampling methods that can identify *all* low-energy regions. We expect that this and other improvements in sampling of the Rosetta energy landscape [47], [48] will help to iteratively improve the energy function until non-native conformations will no longer obtain spuriously low energies.

As expected, Importance Sampling, here in the form of replica exchange Monte Carlo, is more susceptible to spurious low energy regions than the shotgun approach. However, our results also show that this disadvantage is far outweighed by the much improved quality of final ensembles. Moreover, the improved sampling of the new method will allow to thoroughly probe the docking energy landscape, and thus to improve energy functions of both low- and high-resolution stage.

Computationally, the original shotgun approach has the advantage that it runs in an embarrassingly parallel fashion and thus can utilize more computer power in the same period of time than ReplicaDock. Indeed, the ReplicaDock trajectories generated here, required 5 h–72 h of computing on current hardware (using 12 processors), whereas the shotgun sampling can in principle be carried out in a few hours, if thousands of processors are used in parallel. ZDOCK uses an FFT based search of the translational degrees of freedom, rendering it computationally efficient. Only about 1–7 core-hours are required in total, which is <1% of the computational expense for shotgun or ReplicaDock sampling. Additionally, ZDOCK's search could in principle be re-implemented to support an embarrassingly parallel scheme, too. The FFT based search, however, requires grid-based energy functions, and thus is more challenging or even limiting in the possibilities to model interaction energies and to incorporate experimental data.

Despite of the drastic improvement of ReplicaDock to sample low energy structures and to recover near-native basins, there are still a few cases in which ReplicaDock samples very few or even no near-native conformations in the low-resolution stage and thus fails to recover near-native basins after refinement. In these cases, sampling is led astray by alternative binding modes with dramatically increased buried surface area. As it might be difficult or even impossible to ever balance out different contributions to the binding energies, especially at low-resolution and without better treatment of electrostatics (Figure 8), it seems advisable to develop energy functions that are globally flat but locally discriminative, in the sense that well contacting conformations are stabilized regardless their overall buried surface area, whereas miss-aligned conformations with bad shape complementarity are dis-favored. Unfortunately, this is not achieved by simply capping the energy function at a certain cutoff, as this quick fix removes local differences, too. Experimental data, or a higher-resolution energy function, can then be used to discriminate native from non-native conformations.

In this study we benchmarked the performance of replica exchange sampling in RosettaDock. RosettaDock performs a rigid-body minimization followed by all-atom refinement like many other docking programs. The developed method and the conclusions derived from the presented benchmark should thus transfer well to other programs.

## Supporting Information

Supporting Information S1 **This Supporting Information file (PDF) contains supporting Figures S1–S11, Tables S1–S3 and Methods S1–S7.** Figure S1, The distribution of rotation angles. Figure S2, Example of refined decoys overlap with the RelaxedNative ensemble. Figure S3, Automated setup work flow. Figure S4, Detailed analysis of shotgun and ReplicaDock sampling on a bound target 1sq2. Figure S5, Initial survey of temperature selection on bound target 1sq2. Figure S6, Energy distribution of the three temperatures in ReplicaDock for all the benchmark targets. Figure S7, Frequent exchange between bound and unbound state. Figure S8, Different start conformation converge to the same populations. Figure S9, Fraction of hits in low-resolution decoys with different cutoffs. Figure S10, Interface RMSD vs. Interface Energy after refinement of ZDOCK and ReplicaDock ensembles. Figure S11, Interchain_contact dominates docking centroid energy. Table S1, Targets in the benchmark set. Table S2, summary of structure prediction accuracy in unbound docking. Table S3, Average exchange rate between temperature levels across the tested benchmark. Method S1, protocol_capture/rosetta_dock/centroid. Method S2, protocol_capture/replica_dock/centroid. Method S3, protocol_capture/rosetta_dock/refine. Method S4, protocol_capture/replica_dock/refine. Method S5, protocol_capture/relax_native. Method S6, Flags in commandlines in Method S1–S5. Method S7, Automated Setup.
(PDF)
