# Supplementary material for: Replica Exchange Improves Sampling in Low-Resolution Docking Stage of RosettaDock
Source: PLoS One. 2013 Aug 29;8(8):e72096. doi: 10.1371/journal.pone.0072096 (PMC3756964; doi:10.1371/journal.pone.0072096)
Supplement: Supporting Information S1 — This Supporting Information file (PDF) contains supporting Figures S1–S11, Tables S1–S3 and Methods S1–S7. Figure S1, The distribution of rotation angles. Figure S2, Example of refined decoys overlap with the RelaxedNative ensemble. Figure S3, Automated setup work flow. Figure S4, Detailed analysis of shotgun and ReplicaDock sampling on a bound target 1sq2. Figure S5, Initial survey of temperature selection on bound target 1sq2. Figure S6, Energy distribution of the three temperatures in ReplicaDock for all the benchmark targets. Figure S7, Frequent exchange between bound and unbound state. Figure S8, Different start conformation converge to the same populations. Figure S9, Fraction of hits in low-resolution decoys with different cutoffs. Figure S10, Interface RMSD vs. Interface Energy after refinement of ZDOCK and ReplicaDock ensembles. Figure S11, Interchain_contact dominates docking centroid energy. Table S1, Targets in the benchmark set. Table S2, summary of structure prediction accuracy in unbound docking. Table S3, Average exchange rate between temperature levels across the tested benchmark. Method S1, protocol_capture/rosetta_dock/centroid. Method S2, protocol_capture/replica_dock/centroid. Method S3, protocol_capture/rosetta_dock/refine. Method S4, protocol_capture/replica_dock/refine. Method S5, protocol_capture/relax_native. Method S6, Flags in commandlines in Method S1–S5. Method S7, Automated Setup. (PDF) [file pone.0072096.s001.pdf]

Supplement: Replica Exchange improves sampling in low-resolution docking stage of RosettaDock

Zhe Zhang[a], Oliver F. Lange[a,b,*]

a Biomolecular NMR and Munich Center for Integrated Protein Science, Department Chemie, Technische Universität München, 85747 Garching, Germany

b Institute of Structural Biology, Helmholtz Zentrum München, 85764 Neuherberg, Germany

* Corresponding Author:

email: oliver.lange@tum.de;

phone: +49 89 289 13864;

postal: TUM, Chemie, Lichtenbergstrasse 4, 85747 Garching

Key Words: Structure Prediction, Protein Docking, Importance Sampling

Supporting Figures 3
Figure S1: The distribution of rotation angles 3
Figure S2: Example of refined decoys overlap with the RelaxedNative ensemble 4
Figure S3: Automated setup work flow 5
Figure S4: Detailed analysis of shotgun and ReplicaDock sampling on a bound target 1sq2 6
Figure S5: Initial survey of temperature selection on bound target 1sq2 7
Figure S6: Energy distribution of the three temperatures in ReplicaDock for all the benchmark targets 8
Figure S7: Frequent exchange between bound and unbound state. 9
Figure S8: Different start conformation converge to the same populations 10
Figure S9: Fraction of hits in low-resolution decoys with different cutoffs 11
Figure S10: Interface RMSD vs. Interface Energy after refinement of ZDOCK and ReplicaDock ensembles 12
Figure S11: *Interchain_contact* dominates docking centroid energy 13
Support Tables 14
Table S1: Targets in the benchmark set 14
Table S2: summary of structure prediction accuracy in unbound docking 15
Table S3: Average exchange rate between temperature levels across the tested benchmark 17
Supporting Methods 18
Method S1: protocol_capture/rosetta_dock/centroid 18
Method S2: protocol_capture/replica_dock/centroid 18
Method S3: protocol_capture/rosetta_dock/refine 20

Method S4: protocol_capture/replica_dock/refine ... 21
Method S5: protocol_capture/relax_native ... 21
Method S6: Flags in commandlines in Method S1-S5 ... 22
Method S7: Automated Setup ... 22
1) To install: ... 23
2) setup targets library ... 23
3) setup runs ... 23
4) change/add file in /jobtemplates ... 24

Supporting Figures

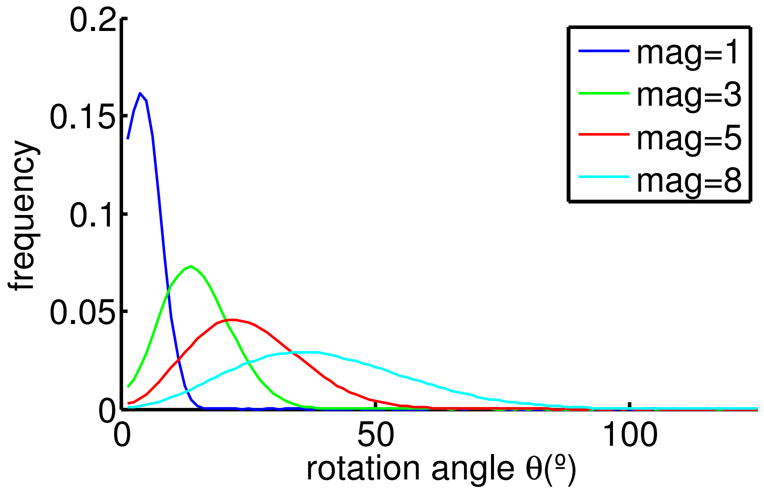

Figure S1: The distribution of rotation angles generated by Rosetta's RigidBodyPerturbNoCenterMover for various "magnitude" parameters. Blue, green, red and cyan correspond to different input parameters of the magnitude.

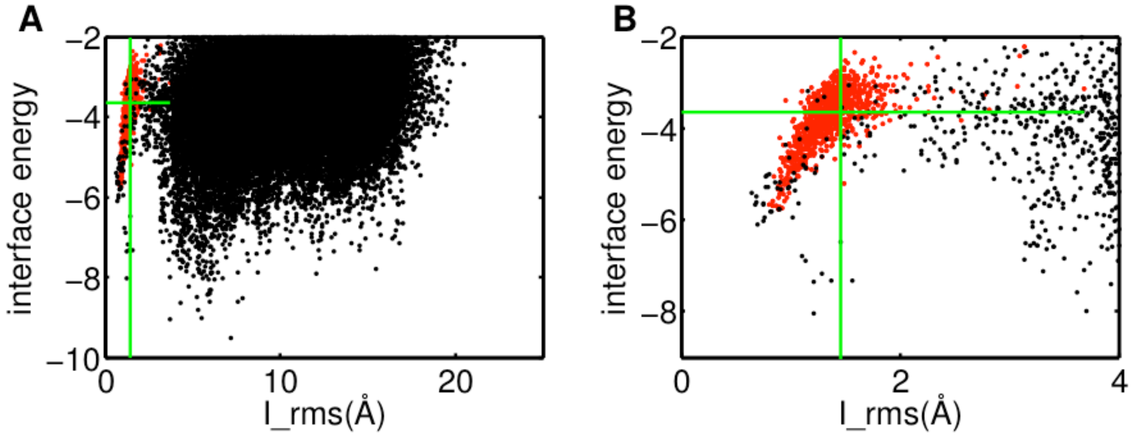

Figure S2: Example of refined decoys overlap with the RelaxedNative ensemble. A) red dots represent the RelaxedNative ensemble, black dots represent decoys refined from centroid decoys generated with one of the three methods shotgun, ZDOCK or ReplicaDock. The green lines define the lower left region and correspond to 50%-tile interface energy and 75%-tile I_rms of RelaxedNatives, respectively. B) zoomed figure of A).

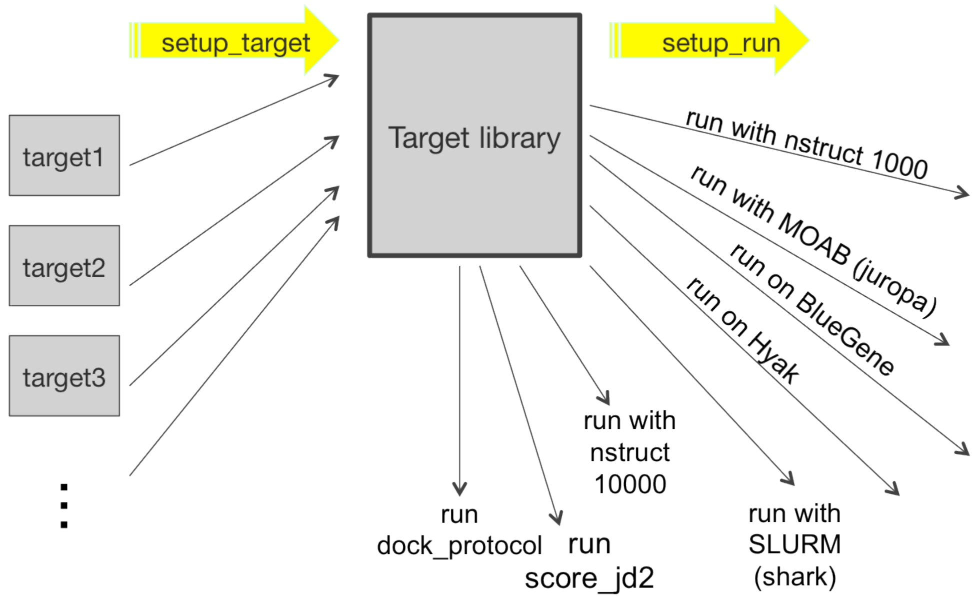

Figure S3: Automated setup work flow.

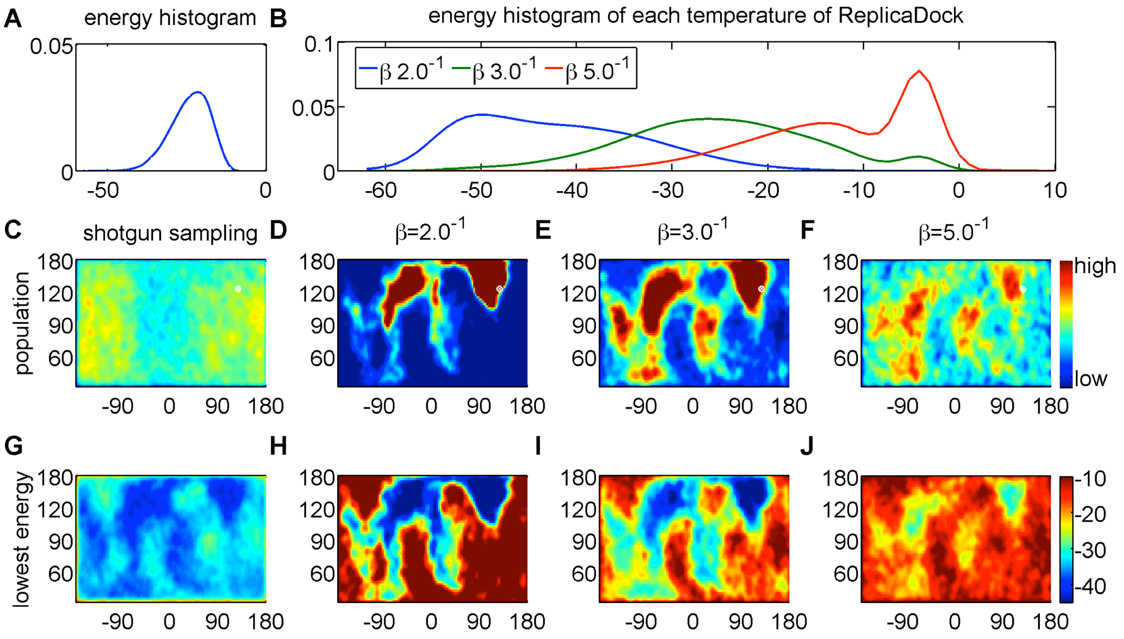

Figure S4: Detailed analysis of shotgun and ReplicaDock sampling on a bound target 1sq2. A) energy distribution of shotgun sampling generated low-resolution decoys. B) energy distribution of conformations sampled by ReplicaDock at respective temperature levels. C-F) Population of sampled conformations in spherical coordinates. Partner A is fixed at the center and the position of Partner B with respect to an idealized spherical surface around Partner A is recorded. The native structure is labeled as white dot (arrow in C). G-J) Conformations are assigned to grid-cells as in C-F, but shown is the lowest energy of all conformations assigned to the respective grid cell. The same color-scale is used for each plot of a row, and the colorbars are attached to the rightmost panel.

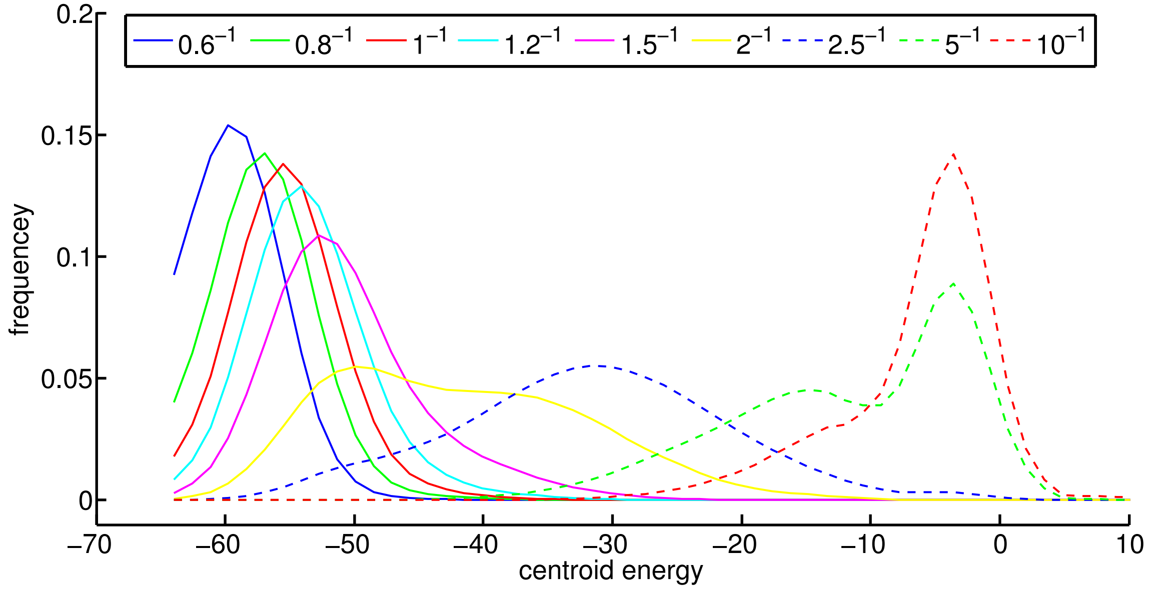

Figure S5: Initial survey of temperature selection on bound target 1sq2. Energy distribution of conformations sampled by ReplicaDock at respective temperature levels.

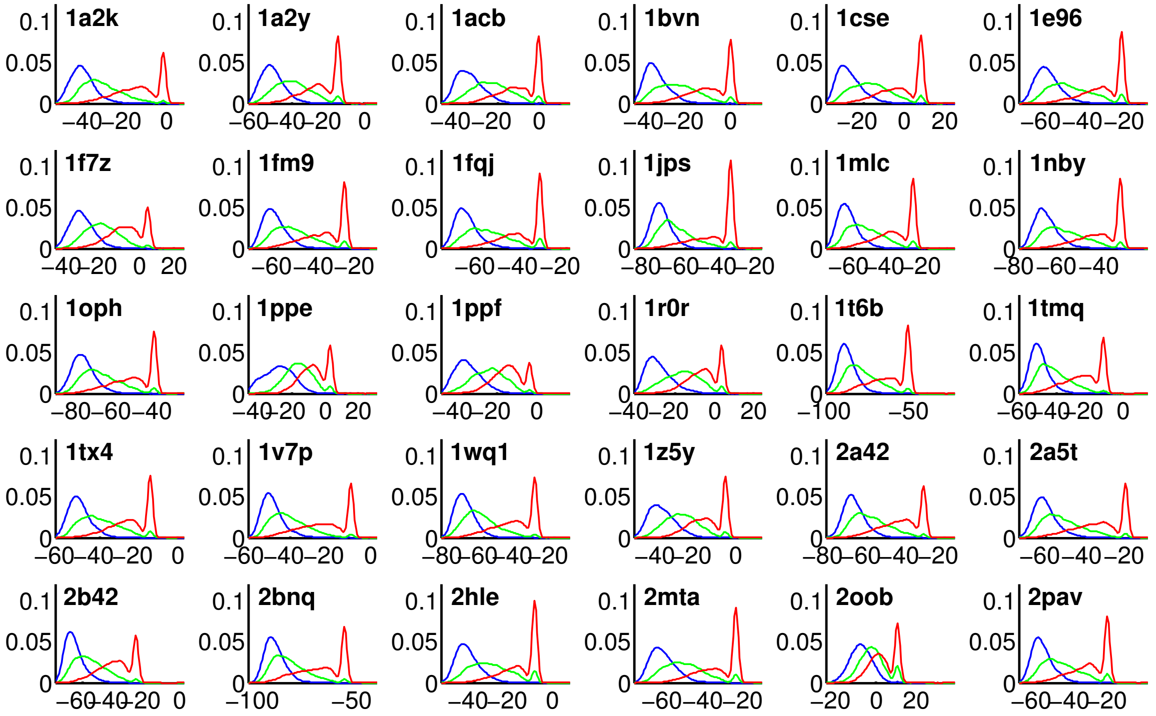

Figure S6: Energy distribution of the three temperatures in ReplicaDock for all the benchmark targets. Blue, green and red correspond to the inverse temperatures $2^{-1}\text{kcal}^{-1}\cdot\text{mol}$, $3^{-1}\text{kcal}^{-1}.\text{mol}$ and $5^{-1}\text{kcal}^{-1}.\text{mol}$. Note that the overall value of the *interchain_env* term is highly target dependent (mainly due to system size), such that the position of the energy-peak reflecting non-contacting conformations changes drastically between targets.

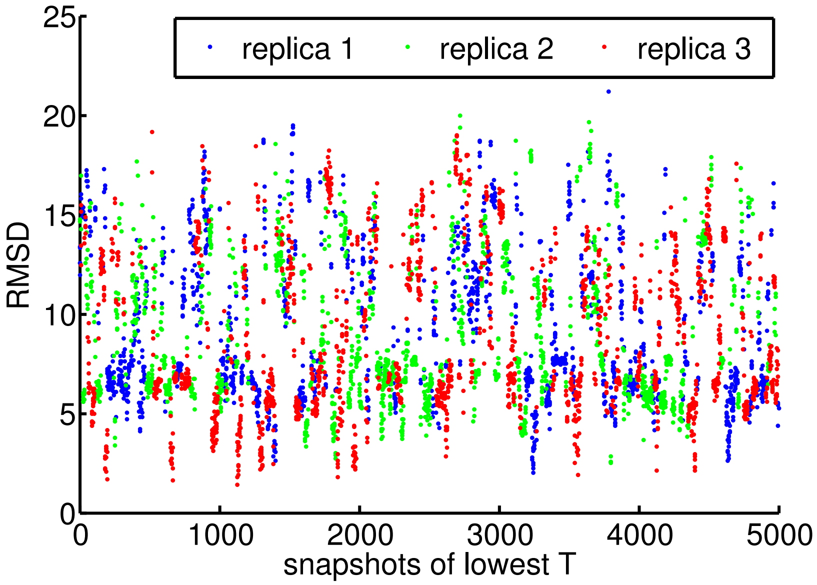

Figure S7: Frequent exchange between bound and unbound state on target 1ppf. RMSD of the snapshots of lowest temperature conformations from the sampled trajectory. 3 colors corresponding to the 3 replicas.

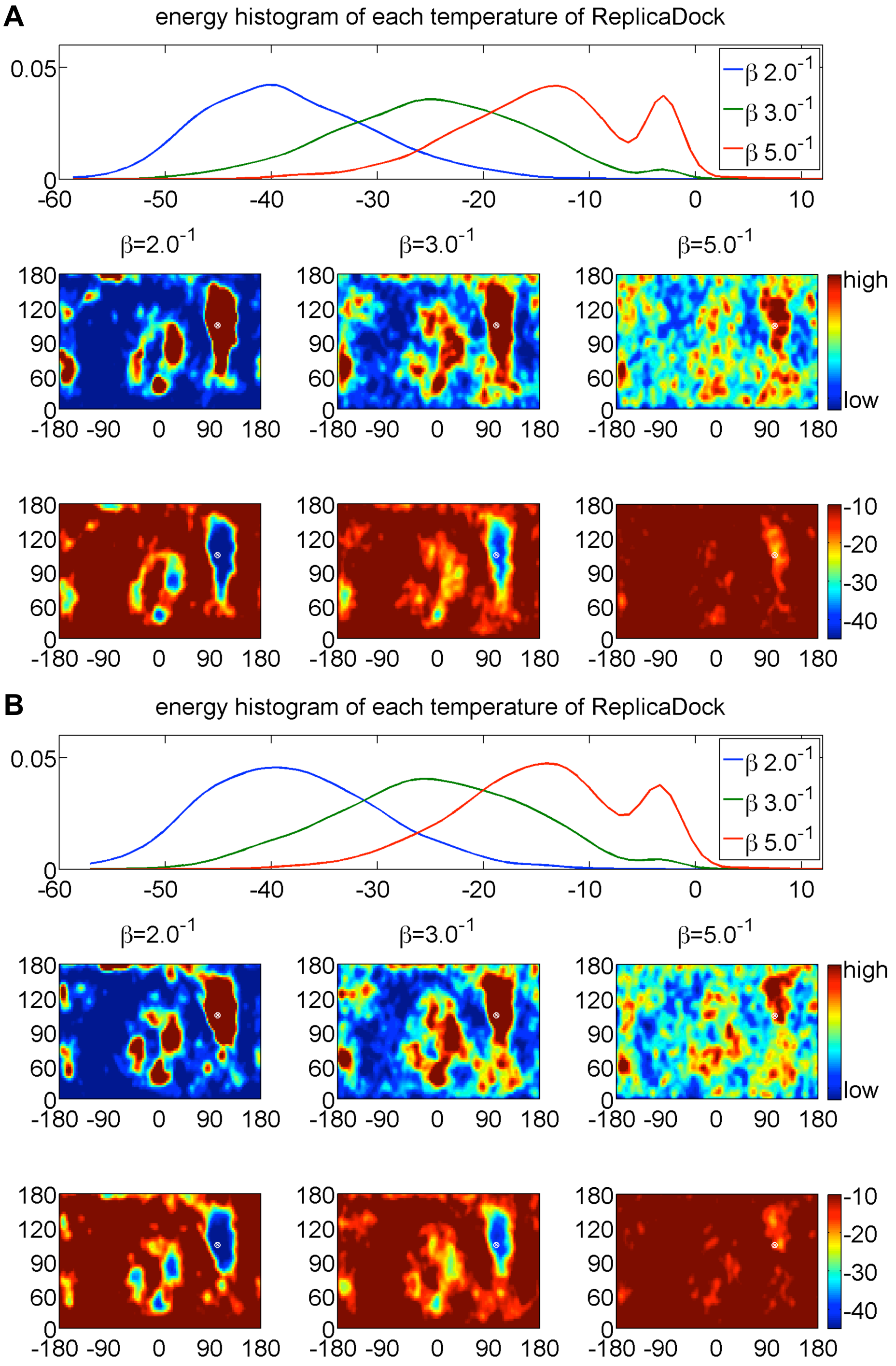

Figure S8: Different start conformations converge to the same populations. Panel A) and B) correspond to ReplicaDock trajectories with different start conformations. Each panel is equivalent to Figure 2 of the main text. (first row) energy distribution of conformations sampled by ReplicaDock at respective temperature level; (second row) population of sampled conformations in spherical coordinates in respective temperature level; (third row) lowest energy of the conformations assigned to each grid.

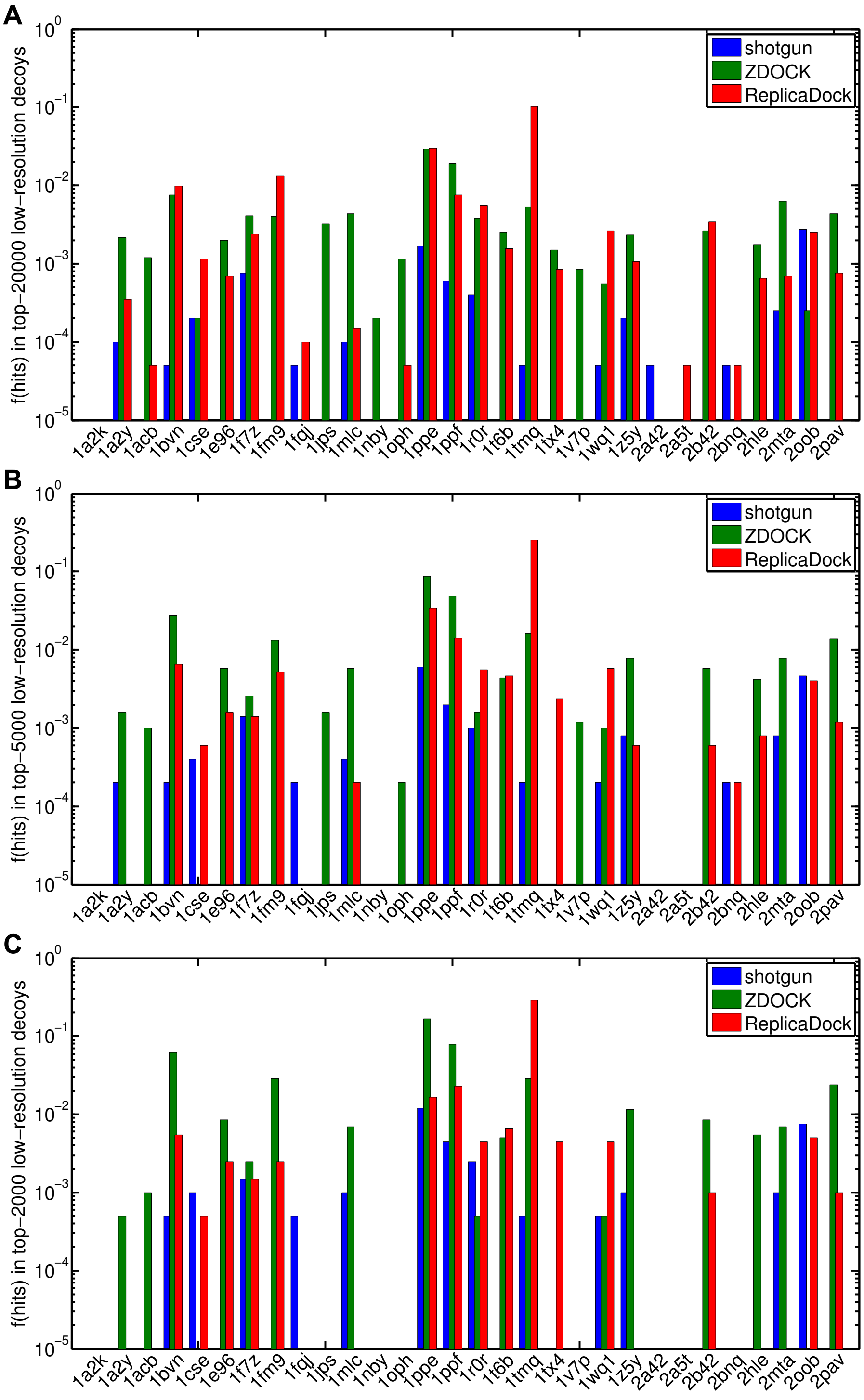

Figure S9: Fraction of hits in low-resolution decoys with different cutoffs. A) Fraction of hits in top-2000 low-resolution decoys, B) Fraction of hits in top-5000 decoys, C) fraction of hits in top-20000 decoys. Blue, green and red correspond to shotgun, ZDOCK and ReplicaDock. Shotgun and ReplicaDock decoys are selected by RosettaDock centroid energy, ZDOCK decoys are selected by ZDOCK score.

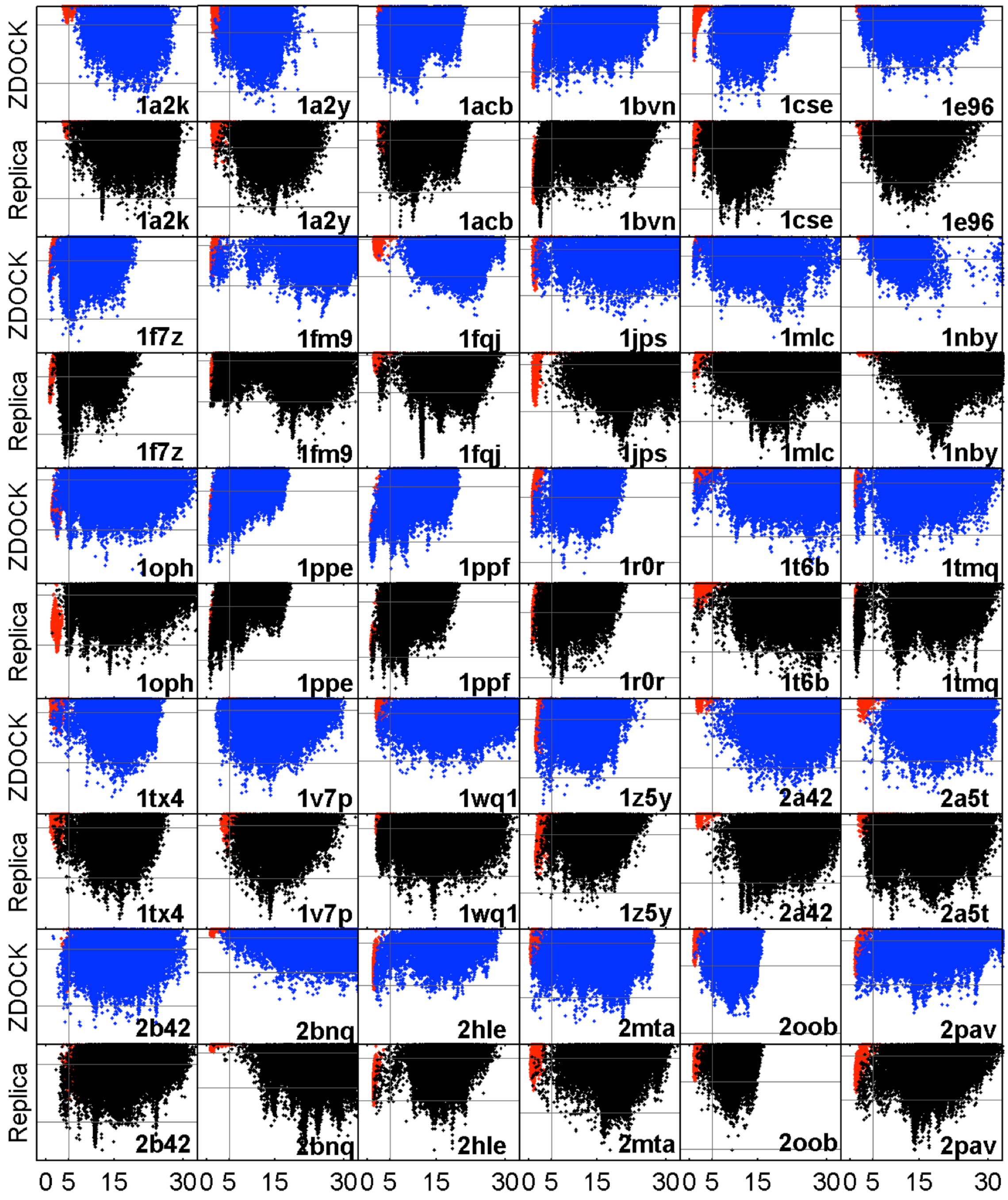

Figure S10: Interface RMSD vs. Interface Energy after refinement of ZDOCK and ReplicaDock ensembles. The red dots represent the RelaxedNative ensembles(Results). The interface RMSD is shown on the x-axis, the interface energy on the y-axis. The same energy range is used for displaying both, ZDOCK (blue) and ReplicaDock (black), results of each target, respectively. The vertical gray lines correspond to I_rms of 5.0 Å, and the two horizontal gray lines correspond to interface energy -4 and -8 Rosetta Energy Units.

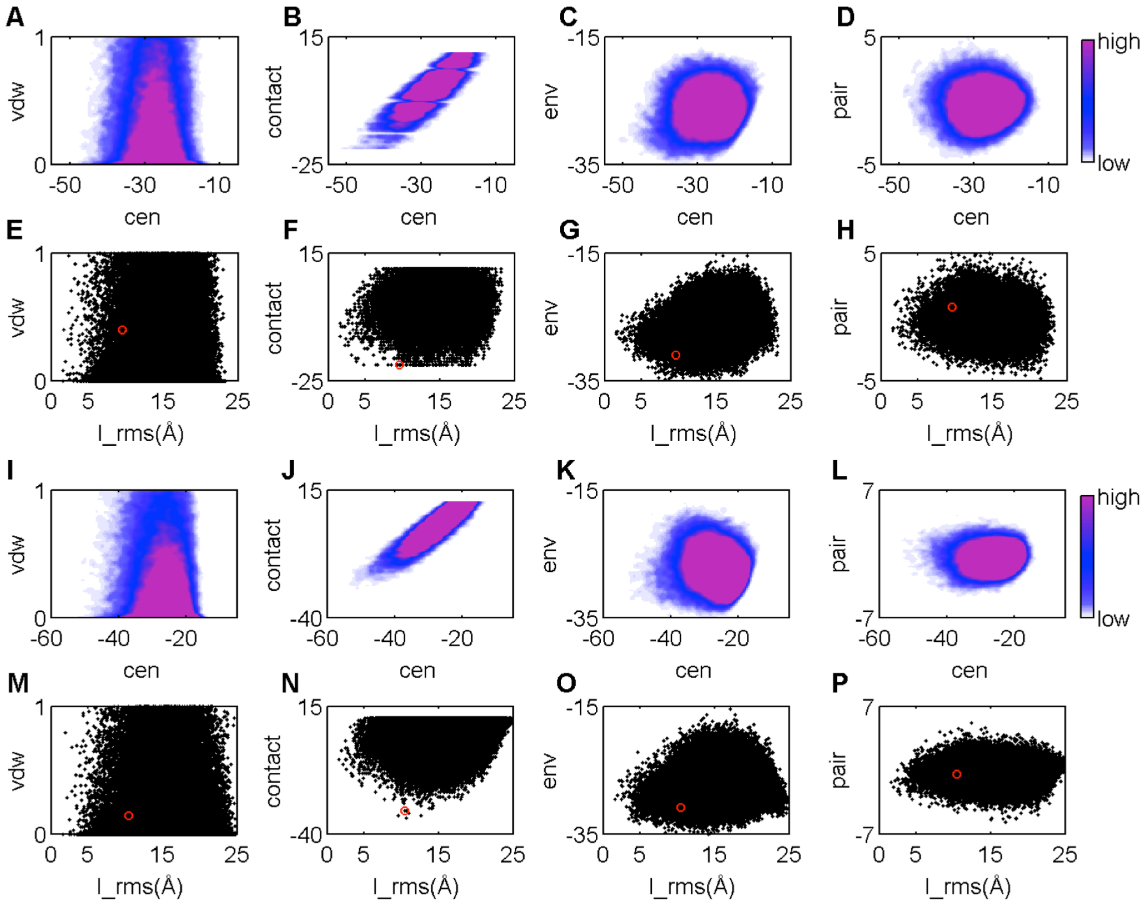

Figure S11: *Interchain_contact* dominates docking centroid energy. A-H) shotgun sampling with capped low-resolution energy function on bound target 1emv, I-P) shotgun sampling without energy capping (Section 3.1). A-D, I-L) total centroid energy versus each individual centroid energy term, i.e. *interchain_vdw, interchain_contact, interchain_env and interchain_pair* in order. E-H, M-P) I_rms versus each individual centroid energy term. The red circle in M-P) corresponding to the denoted decoy in Figure 7 of the main text. The decoy in E-H) denoted as red circle has similar structure to the denoted decoy in Figure 7 of the main text.

Support Tables

1	2	3	4	5	6	7	8	9	10	11	12
1a2k	AB:C	1gy6	AB:AB	100	1.32	1.6	3ran	A:C	99.54	1.32	2.15
1a2y	AB:C	1vfa	AB:AB	100	0.68	1.8	3lzt	A:C	99.22	1.3	0.93
1acb	E:I	1gct	A:E	98.37	0.33	1.6	1egl	A:I	97.78	1.31	2
1bvn	P:T	1hx0	A:P	98.19	0.64	1.38	1hoe	A:T	100	0.45	2
1cse	E:I	1scn	E:E	100	0.3	1.9	1egl	A:I	100	1.21	2
1e96	A:B	1mh1	A:A	98.91	0.72	1.38	1hh8	A:B	99.51	0.63	1.8
1f7z	A:I	1dpo	A:A	99.55	0.33	1.59	5pti	A:I	100	0.32	1
1fm9	D:A	1zgy	A:D	100	1.13	1.8	2p1t	A:A	100	0.74	1.8
1fqj	A:B	1tnd	C:A	91.4	0.54	2.02	1fqi	A:B	100	1.45	1.94
1jps	LH:T	1jpt	LH:LH	100	1.02	1.85	1boy	A:T	100	1.33	2.2
1mlc	AB:E	1mlb	AB:AB	100	0.93	2.1	3lzt	A:E	100	0.75	0.93
1nby	AB:C	1dqq	CD:AB	100	0.73	1.8	3lzt	A:C	99.22	1.44	0.93
1oph	A:B	1qlp	A:A	97.72	1.19	2	2ayw	A:B	99.55	0.33	0.97
1ppe	E:I	1btp	A:E	97.4	0.43	2.2	1lu0	A:I	96.6	0.44	1.03
1ppf	E:I	2rg3	A:E	100	0.39	1.8	2gkr	I:I	100	0.5	1.16
1r0r	E:I	1scn	E:E	100	0.32	1.9	2gkr	I:I	100	0.62	1.16
1t6b	X:Y	1acc	A:X	99.86	1.43	2.1	1shu	X:Y	100	0.59	1.5
1tmq	A:B	1jae	A:A	100	0.39	1.65	1b1u	A:B	99.15	0.98	2.2
1tx4	A:B	1rgp	A:A	99.49	0.69	2	1kmq	A:B	99.43	0.43	1.55
1v7p	AB:C	1ukm	AB:AB	100	0.64	1.9	1dzi	A:C	99.46	0.67	2.1
1wq1	G:R	1wer	A:G	100	0.97	1.6	2ce2	X:R	98.8	1.23	1
1z5y	D:E	1l6p	A:D	99.2	1.15	1.65	2b1k	A:E	99.3	1.06	1.9
2a42	A:B	2fxu	A:A	100	0.82	1.35	2dnj	A:B	100	0.4	2
2a5t	A:B	1pb7	A:A	100	0.59	1.35	2a5s	A:B	100	1.06	1.7
2b42	A:B	1t6e	X:A	99.48	0.56	1.7	1xnb	A:B	100	1.02	1.49
2bnq	DE:AB	2bnu	AB:DE	99.76	1.13	1.4	1i4f	AB:AB	100	1.14	1.4
2hle	A:B	2bba	A:A	100	1.48	1.65	1iko	P:B	100	0.86	1.92
2mta	HL:A	2bbk	JM:HL	97.5	0.37	1.75	2rac	A:A	100	0.62	1.3
2oob	B:A	1yj1	A:B	100	0.87	1.3	2ooa	A:A	100	0.6	1.56
2pav	A:P	2fxu	A:A	100	1.07	1.35	1fil	A:P	100	0.81	2

Table S1: Targets in the benchmark set. Columns 1-2 are co-crystallized structure. Columns 3-7 are unbound structure of the first binding partner, and columns 8-12 are unbound structure of the second binding partner.
Column1: pdb code of co-crystallized structure.
Column 2: interacting chains of co-crystallized structure, separated by colon.
Column 3: pdb code of unbound structure of the first binding partner.
Column 4: chains before colon are in unbound structure, chains after colon are in co-crystallized structure.
Column 5: sequence indentity between unbound and co-crystallized structure of the first binding partner.
Column 6: C_{α}-rmsd of unbound and co-crystallized structure of the first binding partner.
Column 7: crystal structure resolution.
Column 8: pdb code of unbound structure of second binding partner.
Column 9: chains before colon are in unbound structure; chains after colon are in co-crystallized structure.
Column 10: sequence indentity between unbound and co-crystallized structure of second binding partner.
Column 11: C_{α}-rmsd of the unbound and co-crystallized structure of second binding partner.
Column 12: crystal structure resolution.

	shotgun refined					ZDOCK refined					ReplicaDock refined				
	CQ[1]	L_rms	I_rms	f_{nat}	$f_{non\text{-}nat}$	CQ[1]	L_rms	I_rms	f_{nat}	$f_{non\text{-}nat}$	CQ[1]	L_rms	I_rms	f_{nat}	$f_{non\text{-}nat}$
1a2k	0	34.01	14.26	0.021	0.771	0	20.31	11.10	0.000	1.000	0	30.01	12.32	0.146	1.729
1a2y	0	13.98	8.07	0.023	1.023	0	22.91	6.98	0.068	1.455	0	18.13	10.78	0.068	1.045
1acb	0	17.51	7.62	0.014	0.667	*	9.81	3.89	0.145	0.899	**	4.87	2.70	0.333	0.580
1bvn	0	17.78	7.68	0.068	0.753	0	14.73	6.10	0.110	1.014	*	4.72	2.48	0.274	0.781
1cse	0	17.63	7.98	0.013	1.228	0	18.93	7.81	0.000	0.823	*	12.44	3.90	0.241	0.544
1e96	0	31.88	17.76	0.000	1.171	0	27.52	14.56	0.000	1.951	0	27.70	7.01	0.000	1.951
1f7z	0	15.69	5.34	0.098	0.623	0	14.40	3.73	0.066	1.066	0	17.70	4.16	0.197	0.836
1fm9	0	47.93	19.07	0.000	0.682	0	55.13	20.05	0.000	0.727	0	38.02	18.47	0.000	1.136
1fqj	0	31.70	15.74	0.000	1.217	0	31.56	14.68	0.000	1.150	0	27.66	11.89	0.000	2.250
1jps	0	37.20	10.31	0.000	0.972	0	33.54	15.85	0.000	0.986	0	45.33	16.89	0.000	1.155
1mlc	0	22.22	8.80	0.036	0.732	0	26.80	6.88	0.054	0.964	0	56.42	15.58	0.000	2.196
1nby	0	25.56	14.25	0.000	0.811	0	19.95	10.61	0.041	0.757	0	48.01	18.65	0.000	1.311
1oph	0	37.95	12.88	0.000	1.016	0	25.31	5.16	0.032	0.984	0	19.40	4.60	0.145	0.935
1ppe	***	2.65	0.95	0.746	0.085	***	1.76	0.79	0.761	0.197	***	2.11	0.88	0.775	0.113
1ppf	***	2.69	0.94	0.824	0.196	**	3.57	1.12	0.824	0.275	***	2.89	0.99	0.745	0.255
1r0r	0	17.54	6.37	0.000	0.803	*	10.35	2.76	0.394	0.424	0	14.61	4.81	0.167	0.667
1t6b	0	18.23	8.75	0.046	0.969	0	26.39	14.12	0.077	0.615	0	42.47	14.57	0.000	1.169
1tmq	0	19.81	11.88	0.026	0.776	**	4.94	1.43	0.579	0.447	**	4.45	1.36	0.618	0.513
1tx4	0	30.88	13.76	0.000	0.738	0	28.38	12.40	0.031	0.954	0	22.10	11.11	0.062	1.200
1v7p	0	23.22	11.39	0.048	1.048	0	23.99	8.62	0.145	1.081	0	23.53	11.85	0.048	1.290
1wq1	0	22.42	9.62	0.011	0.692	0	16.29	7.67	0.165	0.626	0	11.50	6.87	0.011	1.011
1z5y	*	4.12	1.92	0.273	0.424	*	6.18	3.22	0.333	0.500	0	10.02	4.83	0.288	0.515
2a42	0	37.62	10.93	0.021	1.188	0	43.30	11.67	0.042	1.167	0	43.43	14.97	0.000	1.958
2a5t	0	31.00	16.06	0.034	1.271	0	16.39	8.48	0.017	1.288	0	21.37	10.22	0.000	1.288
2b42	0	29.09	12.70	0.000	0.618	*	9.28	4.15	0.157	0.742	0	17.64	7.31	0.079	0.730
2bnq	0	36.71	14.93	0.000	1.318	0	65.58	24.12	0.000	1.750	0	62.51	24.25	0.000	2.909
2hle	0	24.98	10.70	0.000	0.774	*	6.63	2.91	0.464	0.405	0	36.60	10.28	0.083	0.619
2mta	*	7.25	3.34	0.457	0.630	**	10.11	2.52	0.543	0.565	0	21.06	8.97	0.000	1.174
2oob	0	10.63	5.78	0.111	0.667	0	20.37	5.27	0.074	0.778	0	15.32	9.22	0.000	0.889
2pav	0	18.61	10.74	0.000	0.877	**	3.35	1.45	0.509	0.333	0	46.95	11.90	0.000	1.404
summary	2*/2***					5*/4**/1***					2*/2**/2***				

[1] 'CQ' refers to CAPRI quality

Table S2: summary of structure prediction accuracy in unbound docking. Clusters are ranked by the median of its top 10 interface energies and represented by the lowest interface energy decoy. In column 'CQ' (CAPRI Quality), '0' indicates that none of the top 10 models was of accetable quality, '*', '**' and '***' indicates that at least one of the top 10 models is of acceptable, medium or high quality, respectively (Section 4.7). Columns 'L_rms', 'I_rms', 'f_{nat}' and '$f_{non-nat}$' record the respective information of the best model within these top 10 models.

Target	Exchange rate
1a2k	0.2404
1a2y	0.2394
1acb	0.2504
1bvn	0.2396
1cse	0.2423
1e96	0.2432
1f7z	0.2467
1fm9	0.2578
1fqj	0.2542
1jps	0.2448
1mlc	0.2471
1nby	0.2553
1oph	0.2564
1ppe	0.2687
1ppf	0.2737
1r0r	0.2586
1t6b	0.2566
1tmq	0.2521
1tx4	0.2474
1v7p	0.2582
1wq1	0.2427
1z5y	0.2513
2a42	0.2553
2a5t	0.2515
2b42	0.2494
2bnq	0.2678
2hle	0.2394
2mta	0.2385
2oob	0.3399
2pav	0.2498

Table S3: Average exchange rate between temperature levels across the tested benchmark.

Supporting Methods

Method S1: protocol_capture/rosetta_dock/centroid

```
    # RosettaDock' shotgun approach sampling in low-resolution stage
        # COMMANDLINE:
$ROSETTA3_BIN/docking_protocol.mpi.linuxgccrelease \
-out:file:silent $DECOYS.out
-database $ROSETTA3_DB
-docking:randomize1 -docking:randomize2
-low_res_protocol_only
-nstruct $NSTRUCT
-in:file:native $PROTAB.pdb
-score:weights interchain_cen
-in:file:s $P.pdb
-partners $CHAIN1_$CHAIN2
```

About 120000 decoys for each target are generated in the low-resolution stage with RosettaDock's shotgun approach. For analysis and refinement we take the lowest 36% of decoys by energy and filter out *interchain_contact* > 10. An example decoys file is given in /protocol_capture/2012/replica_docking/example_runs/rosetta_dock/udock_1bvn/run/decoys.out

With this decoy selection would look like this:

```
    # get the tags of the selected decoys. 50 decoys in all in this example
file, so select 50*0.36=18 decoys for analysis and refine
for i in $(ls decoys_000?.out); do echo $i; cat $i | grep SCORE: >> decoys.fsc;
done

scripts/silent_data.py decoys.fsc score interchain_contact description | awk
'$2<=10{print}' | sort -n -k 1 | head -n 18 | awk '{print $3}' > tag_low


    # extract the selected decoys from decoys.out
for i in $(ls decoys_000?.out); do echo $i; scripts/extract_tagged_decoys.py $i
tag_low > low_$i; done
```

Method S2: protocol_capture/replica_dock/centroid

ReplicaDock is run with RosettaScripts. The temperature levels used are 2.0, 3.0 and 5.0. For each target, 4 trajectories with 3 replicas each are run. The trajectory is 5000,000 Monte-Carlo steps and snapshots are stored every 1000 steps. That is for each target ReplicaDock generates 3*4*5e+6/1000=60000 decoys. Decoys with interchain_contact <=10 are selected from the lowest two temperatures (2.0 and 3.0), which makes about 36000 decoys for each targets.

To run ReplicaDock, MPI-mode is required. Please note that specific numbers of processors have to be used: calculate number of processes using the formula: nstruct * n_replica + 2. The extra 2 processes are dedicated to the job distributor and File IO. n_replica is the number of temperature levels (here 3), and nstruct can be any positive

integer. ReplicaDock outputs the trajectory in the form of two silent-files: one containing decoy+score information (name: decoys_<input_pdb>_nnnn_traj.out), and the second file is a copy of just the score information (scores_<input_pdb>_nnnn_traj.out). Decoytags are of the form P_tttt_rrr_sssssss where tttt informs about trajctory number, rrr about the replica, and sssssss about the snapshot number within the trajectory. The temperature levels are switched between different replicas. The current temp_level or temperature of a replica at the moment a decoy was recorded is found in the score-columns *temp_level* and *temperature*.

At the end of a trajectory the final decoy is written to the file 'decoys.out'; this file is a relict of using the JD2-framework and can be ignored. Additionally, the file 'trial.stat' is produced which gives information about acceptance rates in each temperature level.

```
    #  ReplicaDock sampling in low-resolution docking

        # COMMANDLINE:
$ROSETTA3_BIN/rosetta_scripts.mpi.linuxgccrelease \
-database $ROSETTA3_DB
-parser:protocol dock_cen.xml
-n_replica 3
-run:intermediate_structures
-out:file:silent decoys.out             # store final decoys, which can be
                                        # ignored.
-out:file:scorefile scores.fsc
-nstruct 4                              # 4 trajectories running
-partners $CHAIN1_$CHAIN2
-in:file:native $PROTAB.pdb
-in:file:s $P.pdb
-score:weights interchain_cen

    # ReplicaDock SUPPORT_FILES:

        # RosettaScripts file dock_cen.xml
<dock_design>
  <SCOREFXNS>
    <score_dock_low weights="interchain_cen" />
  </SCOREFXNS>
  <FILTERS>
  </FILTERS>
  <MOVERS>

    # sampling in low-resolution stage, thus switch2centroid
    <SwitchResidueTypeSetMover name=switch2centroid set=centroid/>

    # unbiased rigid-body move. Constant parameter for step size, alternatively
use dock_cen_inter.xml, which interpolates the parameter based on temperature
level.
    <ThermodynamicRigidBodyPerturbNoCenter name=rb_mover rot_mag=1
trans_mag=0.5/>

    # setup jumps via fold_tree, store movable_jump into RigidBodyInfo
    <DockSetupMover name=setup_jump/>

    # very loose AtomPair constraint between closest-to-mass-center Ca-atoms
    <AddEncounterConstraintMover name=encounter_cst gap=8/>

    # randomly reorient the two docking partners
    <DockingInitialPerturbation name=init_pert randomize2=1 randomize1=1 />

    # sampling engine

    # acceptance rate recorder, write to file 'trial_stats' at the end
    <TrialCounterObserver name=count file="trial.stats"/>
```

```
    # temp_file contains the temperature configurations. exchange between
neighbor temperatures is attempted every 1000 steps. No specific reason for
using HamiltonianExchange mover instead of ParallelTempering, only because I
started with it and it works as well for temperature only exchange.
    <HamiltonianExchange name=h_exchange temp_file="hamiltonians_cen.txt"
temp_stride=1000/>

    # take snapshots every 1000 steps and write the snapshots into a trajectory
silent file
    <SilentTrajectoryRecorder name=traj score_stride=1 stride=1000
cumulate_replicas=1 />

    # normally use trials=5000,000. Empirically, it is enough for converge.
    <MetropolisHastings name=sampler trials=5000000 scorefxn=score_dock_low >
      <Add mover_name=h_exchange/>
      <Add mover_name=traj/>
      <Add mover_name=count/>
      <Add mover_name=rb_mover/>
    </MetropolisHastings>

  </MOVERS>
  <APPLY_TO_POSE>
  </APPLY_TO_POSE>
  <PROTOCOLS>
    <Add mover_name=switch2centroid/>
    <Add mover_name=setup_jump/>
    <Add mover_name=encounter_cst/>
    <Add mover_name=init_pert/>
    <Add mover_name=sampler/>
  </PROTOCOLS>
</dock_design>


    # temperatures configuration file "hamiltonians_cen.txt"
GRID_DIM 1
GLOBAL_PATCH atom_pair_constraint = 5   # set atom_pair_constraint weight to 5
                                        # for all replicas
1 2.0 interchain_cen                    # define temp and score of replicas
2 3.0 interchain_cen
3 5.0 interchain_cen
```

For analysis and refinement, decoys are selected as follows:

```
    # get the tags of the selected snapshots
cat scores_P_000*fsc > scores_traj.fsc

scripts/silent_data.py scores_traj.fsc temperature interchain_contact
description | awk '$1<4&&$2<=10{print $3}' > tag_low

    # extract selected decoys from the trajectory silent file
for i in $(ls decoys_P_000?_traj.out); do echo $i;
scripts/extract_tagged_decoys.py $i tag_low > low_$i; done
```

Method S3: protocol_capture/rosetta_dock/refine

Refinement of low-resolution ensemble is carried out with standard parameters. If disulfide bonds are present in the unbound structures, extra flags are added:

```
    -detect_disulf  true -rebuild_disulf  true  -fix_disulf  $DISULF_FILE

    # COMMANDLINE:
$ROSETTA3_BIN/docking_protocol.mpi.linuxgccrelease \
-database $ROSETTA3_DB
-evaluation:rmsd IRMS _input FULL
-in:file:native $PROTAB.pdb
```

```
-docking_local_refine
-ex1
-ex2aro
-nstruct 1
-score:weights docking
-use_input_sc
-unboundrot $PROTAB.pdb

-detect_disulf true                  # these three disulf related flags used
-rebuild_disulf true                 # only if disulfide bonds are present
-fix_disulf $DISULF_FILE             # in the unbound structures

-in:file:silent $SELECTED_LOW_RES.out
-out:file:silent $REFINED.out
```

Method S4: protocol_capture/replica_dock/refine

Refinement of low-resolution ensembles from ReplicaDock as well as ReplicaDock-LoT are done using exactly the same protocol as refinement of low-resolution ensemble from shotgun approach.

Method S5: protocol_capture/relax_native

Using the same refinement protocol as for refinement of low-resolution ensembles, we generated the RelaxedNative ensembles starting from the superimposed reference structure with 1000 decoys for each target.

```
    # COMMANDLINE:
$ROSETTA3_BIN/docking_protocol.mpi.linuxgccrelease \
-database $ROSETTA3_DB
-evaluation:rmsd IRMS _input FULL
-in:file:native $PROTAB.pdb
-docking_local_refine
-ex1
-ex2aro
-nstruct 1000                     # generate 1000 decoys as RelaxedNative for
                                  # each target
-score:weights docking
-use_input_sc
-unboundrot $PROTAB.pdb

-detect_disulf true
-rebuild_disulf true
-fix_disulf $DISULF_FILE

-in:file:s $PROTAB.pdb            # start from the superimposed structure
-out:file:silent $REFINED.out
```

Method S6: Flags in commandlines in Method S1-S5

Here we list out the explanations for the flags mentioned in Method S1-S5 as follows.

flags	interpretation
-out:file:silent $DECOYS.out	specify the filename of output silent file
-database $ROSETTA3_DB	specify the directory of your rosetta database
-docking:randomize1 -docking:randomize2	randomize1 and randomize2 using together to do global docking
-low_res_protocol_only	low-resolution stage only with RosettaDock --- shotgun sampling
-nstruct $NSTRUCT	specify how many decoys to generate
-score:weights interchain_cen	centroid docking energy function
-partners $PARTNER1_$PARTNER2	specify the docking partners, for example "AB_C" means docking chain C to chain AB, and keep chain AB rigid
-evaluation:rmsd IMRS_input FULL	evaluate the Ca-RMSD for the entire structure
-docking_local_refine	only do refinement with RosettaDock
-ex1 -ex2aro	adding extra sidechain rotamers
-use_input_sc	Use accepted rotamers from the input structure between Monte-Carlo with Minimization (MCM) cycle
-unboundrot $PROTAB.pdb	use unbound rotamers
-detect_disulf true -rebuild_disulf true -fix_disulf $DISULF_FILE	these three flags are added if disulfide bonds are present in the unbound structures. in $DISULF_FILE residue pairs forming disulfide bonds are specified, like "28 35"
$TARGETLIB	protocol_capture/2012/replica_docking/dock_targetlib
$PROTAB.pdb	PDB-file with unbound partners superimposed onto the bound complex, used as the reference structure for RMSD related calculation
$P.pdb	PDB-file with the two binding partners in $PROTAB.pdb fully randomized, used as the start conformation to avoid initial bias
-n_replica	number of replicas, need to be consistent with the number of temperature levels in file hamiltonians_cen*.txt

Method S7: Automated Setup

All the production runs of the benchmark in this work are generated with the automated setup tools available with the CS-Rosetta toolbox(www.csrosetta.org). The workflow of this automated setup is shown in Figure S12. Methods for docking used in this work are included in the folder under *protocol_capture/replica_docking/csrosetta3/flag_library/methods/*, i.e. *_docking_base, rosetta_dock* and *replica_dock*. To run this toolbox, a minmal installation is required as detailed below.

1) To install:

```
    # go to the directory
cd /csrosetta3/

    # check all the options available for the installation
install.py -h

    # use option -nopicking since fragment picking is not required for docking,
and provide path to ROSETTA as follows
install.py -nopicking -rosetta ~/rosetta -rosetta_database
~/rosetta/rosetta_database

    # You will see some installation information. Finally you should see
'created symlink /home/zhezhang/csrosetta3/com/init.bashrc -->
/home/zhezhang/csrosetta3/com/init' with /home/zhezhang/ replaced by your own
user directory. To use the toolbox in your shell run
source /home/zhezhang/csrosetta3/com/init
```

2) setup targets library

This step assembles target related input files to build the target library (Figure S12). By default the library is stored in ~/cs_targetlib. You can also specify a directory using flag '-target_prefix' as follows. Absolute path is recommended for '-target_prefix'.

```
    # Setup a target for rosetta_dock
setup_target -method rosetta_dock -target udock_1bvn -target_prefix
$TARGETLIB_DIR -disulf disulf_file -native protAB.pdb -pdb P.pdb -partners
partners

    # Setup a target for replica_dock either explicitly using
setup_target -method replica_dock -target udock_1bvn -target_prefix
$TARGETLIB_DIR -native protAB.pdb -pdb P.pdb -partners partners

    # or copying the inputs from a previously prepared 'rosetta_dock' setup as
follows:
setup_target -method replica_dock -target udock_1bvn -target_prefix
$TARGETLIB_DIR -transfer_method rosetta_dock
```

3) setup runs

This step creates a run-ready directory as specified with flag '-dir', in which job-scripts, input files, RosettaScripts-xml as well as flag files are contained. For flag '-dir', absolute path is recommended. For job scripts, you can use different types (e.g. moab) according to your queuing system, as shown in Figure S12. I will keep slurm (-job slurm) here as example.

For single-machine/interactive use, you can simply specify with flag '*-job interactive*' when setup the run, then start the running under the corresponding run/ directory using '*source production.interactive.job -n $Np*' with *$Np* specifying the processor numbers.

```
    # setup a run of ReplicaDock in queuing system
setup_run -method replica_dock -target udock_1bvn -target_prefix $TARGETLIB_DIR
-dir $TEST_REPLICA_DIR -job slurm -extras mpi -score interchain_cen -nstruct 1
-protocol rep_cen -xml uniform -n_replica 3

    # rosetta_dock's shotgun sampling in low-resolution stage
setup_run -method rosetta_dock -target udock_1bvn -target_prefix $TARGETLIB_DIR
-dir $TEST_ROSETTA_DIR -job slurm -extras mpi -protocol centroid -batches 2
-score interchain_cen -nstruct 100

    # refine decoys sets
setup_run -method rosetta_dock -target udock_1bvn -target_prefix $TARGETLIB_DIR
-dir $TEST_REFINE_DIR -job slurm -extras mpi -protocol refine -pattern
```

```
"low_decoys_*out" -prefix refine -score docking -nstruct 1 -start
$ABSOLUTE_PATH_OF_FOLDER_FOR_DECOYS

   # refine protAB.pdb to generate relaxNative ensemble
setup_run -method rosetta_dock -target udock_1bvn -target_prefix $TARGETLIB_DIR
-dir $TEST_RELAX_NATIVE_DIR -job slurm -extras mpi -protocol refine -out
relax_native.out -score docking -nstruct 1000
```

4) change/add file in /jobtemplates

You can easily modify the jobtemplates for use with your own queuing system. Shown here is a job template for slurm. The $CM_ variables are replaced by the automatic setup tool when run setup_run. #SBATCH and $SLURM_ are specific to SLURM queuing environment.

```
#!/bin/bash -x
#SBATCH -J csrosetta
### start of jobscript

NSLOTS=$SLURM_NTASKS

module load openmpi/gcc

LOGS=logs_`echo $SLURM_JOB_ID | awk -v FS="." '{print $1}'`
mkdir -p $LOGS

## have NSLOTS - 3 worker processes -- determines number of structures per
generation...
NSTRUCT=`echo $NSLOTS | awk '{print $1-3}'`

echo "running on $NSLOTS cpus ..."
EXE=$CM_EXECUTEABLE.$CM_EXEC_EXT
CMDLINE="-out:level 300 -mute all_high_mpi_rank_filebuf -out:mpi_tracer_to_file
$LOGS/log -database $CM_ROSETTA_DATABASE $CM_COMMANDLINE"
CYCLES=$CM_AUTO_NSTRUCT
$MPI_RUN -n $NSLOTS $EXE $CMDLINE $CYCLES
```
